# A deep learning model to classify and detect brain abnormalities in portable microwave based imaging system

**DOI:** 10.1038/s41598-022-10309-6

**Published:** 2022-04-15

**Authors:** Amran Hossain, Mohammad Tariqul Islam, Ali F. Almutairi

**Affiliations:** 1 Department of Electrical, Electronic and Systems Engineering, Faculty of Engineering and Built Environment, Universiti Kebangsaan Malaysia, 43600 Bangi, Malaysia; 2 Department of Computer Science and Engineering, Dhaka University of Engineering & Technology, Gazipur, Gazipur, 1707 Bangladesh; 3 Electrical Engineering Department, Kuwait University, 13060 Kuwait City, Kuwait

**Keywords:** Biological physics, Cancer imaging, Imaging and sensing

## Abstract

Automated classification and detection of brain abnormalities like a tumor(s) in reconstructed microwave (RMW) brain images are essential for medical application investigation and monitoring disease progression. This paper presents the automatic classification and detection of human brain abnormalities through the deep learning-based YOLOv5 object detection model in a portable microwave head imaging system (MWHI). Initially, four hundred RMW image samples, including non-tumor and tumor(s) in different locations are collected from the implemented MWHI system. The RMW image dimension is 640 × 640 pixels. After that, image pre-processing and augmentation techniques are applied to generate the training dataset, consisting of 4400 images. Later, 80% of images are used to train the models, and 20% are used for testing. Later, from the 80% training dataset, 20% are utilized to validate the models. The detection and classification performances are evaluated by three variations of the YOLOv5 model: YOLOv5s, YOLOv5m, and YOLOv5l. It is investigated that the YOLOv5l model performed better compared to YOLOv5s, YOLOv5m, and state-of-the-art object detection models. The achieved accuracy, precision, sensitivity, specificity, F1-score, mean average precision (mAP), and classification loss are 96.32%, 95.17%, 94.98%, 95.28%, 95.53%, 96.12%, and 0.0130, respectively for the YOLOv5l model. The YOLOv5l model automatically detected tumor(s) accurately with a predicted bounding box including objectness score in RMW images and classified the tumors into benign and malignant classes. So, the YOLOv5l object detection model can be reliable for automatic tumor(s) detection and classification in a portable microwave brain imaging system as a real-time application.

## Introduction

Worldwide, brain abnormalities such as tumors are the prime reasons for fatality and incapacity since it damages the primary cell of the human brain. A brain tumor enhances abnormal tissues that create inside the human head. Due to the brain abnormality effectiveness, the possibility of brain cancer in the head is increasing day by day, and it is the 9th dominant reason of death for women, men, and children^1^. Brain abnormalities might be fatal, crucially affecting the quality of longevity and changing the whole thing for patients and their families. Brain tumor as an abnormality is mainly classified into two categories: benign and malignant tumor^2^. The benign tumors are non-cancerous cells and have a homogeneous structure with a regular shape. In contrast, malignant tumors are cancerous cells and have a heterogeneous structure with an irregular shape^1^. In addition, benign tumors have a slower growth rate, and malignant tumors can grow uncontrollably. The percentage of death rate increases due to the invasive features of the tumors. But, early detection of brain tumors might increase the survival rate of a human. At present, different modern imaging modalities: PET(positron emission tomography), X-ray screening, MRI (magnetic resonance imaging), CT (computed tomography) scans, and ultrasound screening are used to identify brain tumors in medical applications^2–4^. The key disadvantages of the conventional medical imaging modalities are increasing cancerous risk due to high dose radiation, lower susceptibility, high ionizing with brain cells, costly, and danger for the old patient and pregnant women^2,5–10^. In the last decades, microwave imaging (MI) research has been growing in medical applications due to its abundant properties: non-ionizing radiation, inexpensive, non-invasive, and safe for the human body compared to conventional medical imaging modalities^11–17^. A microwave imaging system consists of an antenna array, where the transmitting antenna produces the microwaves and transmits them to the target objects. The receiving antennas receive the backscattering waves. After that, the image reconstruction algorithm is applied to the collected scattering signals to generate the reconstructed microwave image, including the target object of interest (ROI). A wideband antenna is indispensable in a microwave head imaging (MWHI) system that works within the frequency band of 1 GHz to 4 GHz with unidirectional radiation characteristics, high gain, and high efficiency^11,18–24^. Various MWHI systems have been implemented over time by using multiple types of two-dimensional (2D)^15,18,23–28^ and three-dimensional(3D) antenna arrays to identify the brain tumor^14,19,22,29–32^.

Recently, the deep neural networks (DNNs) technique has been applied in medical imaging to identify brain abnormalities due to the benefit of making decisions with significantly less involvement from human interaction. The DNN filters the imaging data through the cascade of multiple layers and provides a more accurate result as an output, and it is faster than the traditional imaging system^33–36^. Researchers are currently applying the DNN technique in microwave imaging applications to identify the target object and imaging purposes^36–42^. An autoencoder-based deep learning method was investigated to reconstruct the microwave image of the different shaped objects^40^. The technique used a 24 × 24 antenna array system, which generated EM signals and propagated through the network to reconstruct the image. But no object detection mechanism was presented in the article. A convolutional neural network(CNN) based U-Net segmentation framework was used for masking the tumor object in MW imaging^41^. The framework was investigated on only the simulated data but did not implement any experimental framework and detection algorithm. An automatic DNN based classification and detection framework was presented in^43^. The approach classified only the tumor and non-tumor images and then masked the image's tumor regions. But the process is failed to classify the benign or malignant tumor in the images. A faster region-based CNN (Faster R-CNN) model was used to detect and classify brain tumors^44^. The outperforms of the model were comparatively low due to the small training dataset. It achieved 91% classification accuracy, and it failed to classify the small tumor in the images. The Mask R-CNN model^45^ improved the tumor detection accuracy. This approach improved the overall detection accuracy but failed to detect and classify malignant tumors in the images due to diverse tumor profiles. A handcrafted deep learning feature-based brain tumor detection approach was presented in^46^. The approach used the grab cut technique to segment the tumor regions, and then hand crafted including deep features was used to diminish the noise level in the image, but the abnormalities classification and specific tumor detection scenarios were not presented in the literature. A fully CNN (F-CNN) method was applied to the brain tumor image dataset to detect the brain abnormalities^47^. The technique was used U-Net segmentation scheme with an adam optimizer algorithm to segment the tumor region in the images. This approach able to identify the tumor, but tracing the appropriate location and classification of the tumor is difficult because of the shortage of impartial predictors. Rresidual CNN (R-CNN) was applied to the brain images to recognize and classify the tumors in the brain images^34^. The method used data augmentation and distillation procedures to mitigate the unwanted signal in the image, but tumor identification setup with exploratory analysis was not accomplished in the article. A DNN network-based super-resolution algorithm (SRA) was proposed for MW imaging purposes^48^. The network used a 33 × 33 image dataset to train the model, and it produced two-dimensional images. But it was seen that, the produced images were noisy, blurry, and the accuracy of classification was very low due to the small training dataset. Thus, detecting the precise position of the target tumor in the images was complicated, and the approach failed to classify the benign and malignant tumor. A YOLOv3 deep learning model was used to identify and localize the tumor with bounding box in microwave images^14^. The model detected the tumors, but failed to classify the benign and malignant tumor(s) in reconstructed images.

It is observed that the stated MWHI systems used numerous traditional microwave imaging algorithms to identify the target tumor from the reconstructed images. It is problematic to detect the precise position of the tumor because of the noisy, irregular tumor shape and low-quality image resolution. In addition, benign and malignant tumor classification is more challenging in real-time applications. As a result, it is necessary to manually indicate the locations and classify the tumor(s) in the reconstructed images by the professional physician or technician, which is a vigorous problem of the traditional MWHI system. Therefore, the inspiration of the research to mitigate the issues in the stated MWHI scheme relies on an object detection algorithm is required in the real application that can automatically detect the tumor(s) with precise location and classify them into two classes (i.e., benign and malignant) in the reconstructed microwave brain images. The key contributions of the research work might be specified below:

(i) According to the author's knowledge, it is the first research work where a YOLOv5 deep neural network-based object detection model is employed in a portable microwave brain imaging system as a real-time application due to its abundant features. The YOLOv5 model can automatically detect small and large sized brain tumor(s) with a bounding box including objectness score and classify the brain tumors into two classes: benign and malignant.
(ii) A 3D wideband antenna array is utilized in the implemented imaging framework and investigated by the fabricated tissue-mimicking head phantom, including a benign and a malignant tumor, for generating reconstructed microwave (RMW) brain images.
(iii) Implemented the YOLOv5 model with its three versions: YOLOv5s, YOLOv5m, and YOLOv5l to investigate the detection and classification performances by utilizing training and testing datasets.
(iv) The performances of the YOLOv5l model are compared with five other state-of-the-art object detection models: Faster R-CNN, Mask R-CNN, F-CNN, Residual R-CNN, and YOLOv3.
(v) The detection performance is investigated and compared with the fabricated phantom images and simulated images as an alternative to collected in-vivo images to ensure real application accuracy.

In this research, we have developed a portable microwave brain imaging system to produce microwave brain images, which can be used in the rural area as a real application due to its safety and portability features with low cost. It is noticeable that early diagnosis, automatic tumor identification with location, and classification are crucial without an object detection algorithm when the system is integrated with the high-end computational device. The overall system needs low computational inference time, automatic detection characteristics with objectness score including predicted bounding box, and high classification performances. A deep learning-based object detection algorithm can be a reliable solution to overcome these issues. YOLOv5 is a popular single-stage deep learning algorithm that uses CNN for object detection. This model reduces inference speed, increases object detection accuracy, and enhances localization accuracy^49^. The traditional deep learning algorithms are incapable of identifying an object in a single run, but the YOLOv5 makes the detection in a single run through the NN (neural network), which makes it suitable for real-time application and high-end computational devices. Thus, we have been used YOLOv5 as a classifier instead of the conventional deep convolutional neural network to get significant benefits in our research. The significant benefits of the YOLOv5 in our work are: (i) it has a lightweight architecture with low computational complexity, (ii) it takes low inference time to update the weights which properly train the model, (iii) it has three scaling features maps to predict, localize the small, medium, and large sized object (i.e., tumor) with a predicting bounding box, (iv) it shows the objectness score of the predicting tumor object, that help to improve the tumor classification performances, and (v) it can monitor and identify the target tumor in noisy, blurry, and low resolution based RMW brain images.

The overall process is discussed as follows: initially, the RMW image samples are collected from the implemented MWHI system. The RMW image dimension is 640 × 640 pixels. Four hundred image samples with non-tumor and tumor in different locations are collected from the implemented MWHI system. After that, image pre-processing and augmentation techniques are applied to generate the training dataset. The training dataset consists of 4400 images, including single benign and malignant tumors, double benign tumors, double malignant tumors, single benign and single malignant tumors. Later, 80% of images are used to train the model, and 20% are used for testing. From the 80% training dataset, 20% are utilized to validate the models. The YOLOv5 model with its three versions (YOLOv5s, YOLOv5m, and YOLOv5l) is implemented and executed by the Python high-level language with a powerful PyTorch machine learning library. The tumor classification and detection performances are investigated on various datasets by the YOLOv5s, YOLOv5m, and YOLOv5l models. In addition, training vs. validation loss, training classification loss vs. validation classification loss, and training vs. validation accuracy are investigated to evaluate the models. Moreover, the precision, recall, specificity, F1-score, and mean average precision (mAP) are observed to verify the models. It is seen that the YOLOv5l performed better than the other two models. The training accuracy, validation loss, precision, recall, specificity, F1 score, train classification loss, validation classification loss, and mAP for YOLOv5l model are 99.84%, 9.38%, 93.20%, 94.80%, 94.21%, 94.00%, 0.004, 0.0133, and 96.20% respectively. It is noticeable that the lower validation loss, higher accuracy, precision, recall, specificity, F1 score, and lower train and validation classification losses confirm the YOLOv5l shown better detection and classification performances. Also, the detection and classification outcomes are evaluated by the YOLOv5s, YOLOv5m, and YOLOv5l models. Then the YOLOv5l model is compared with other state-of-the-art object detection models. It concludes that the YOLOv5l model can be reliable for automatic tumor detection and classification in microwave brain imaging systems.

The remaining part of the article is structured as follows: Experimental investigation of the MWHI system is illustrated in section “Experimental investigation of MWHI system”. A comprehensive study of the deep learning-based YOLOv5 object detection model with its background is discussed in section “Background analysis of YOLOv5 model”. Overall research methodology is presented in section “Classification and detection methodology and materials”. A detailed explanation of the training experiments is discussed in section “Details explanation of the training experiments”. Afterward, the results and discussions are explained in section “Result and discussion” and concluded in section “Conclusion”.

## Experimental investigation of MWHI system

**Figure 1:**
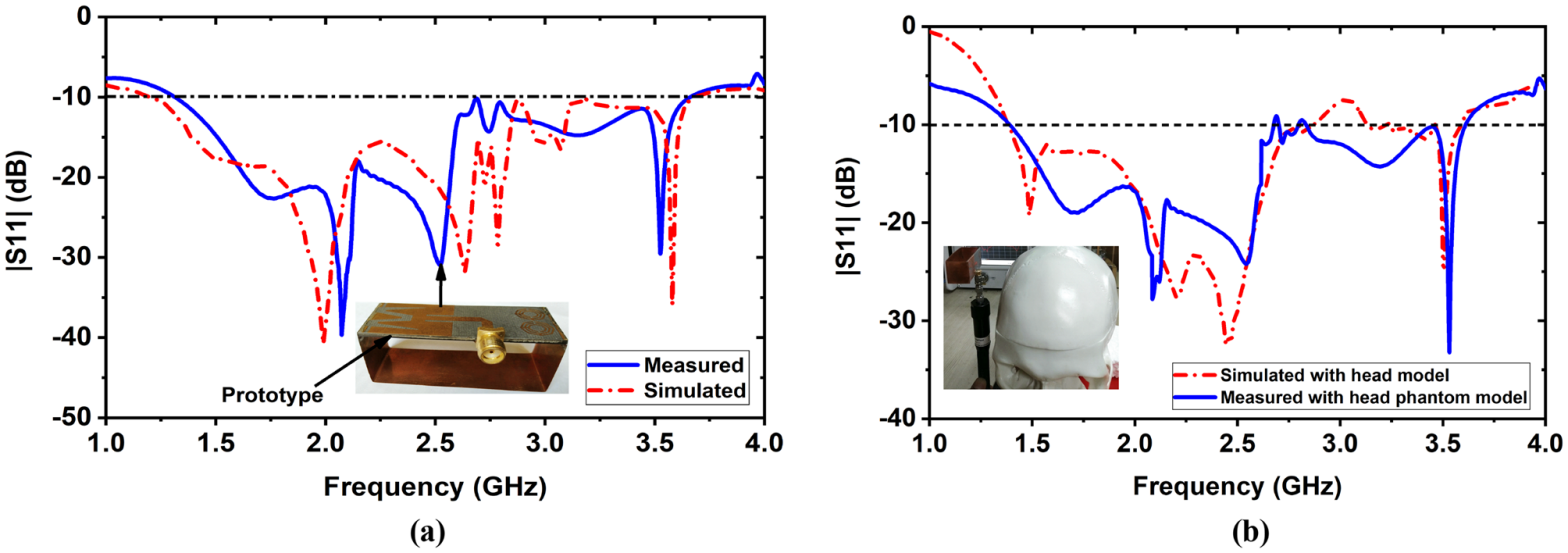
Measured and simulated outcomes of the 3D antenna: (**a**) |S11| parameters in open space, (**b**) |S11| parameters with the head phantom model.

**Figure 2:**
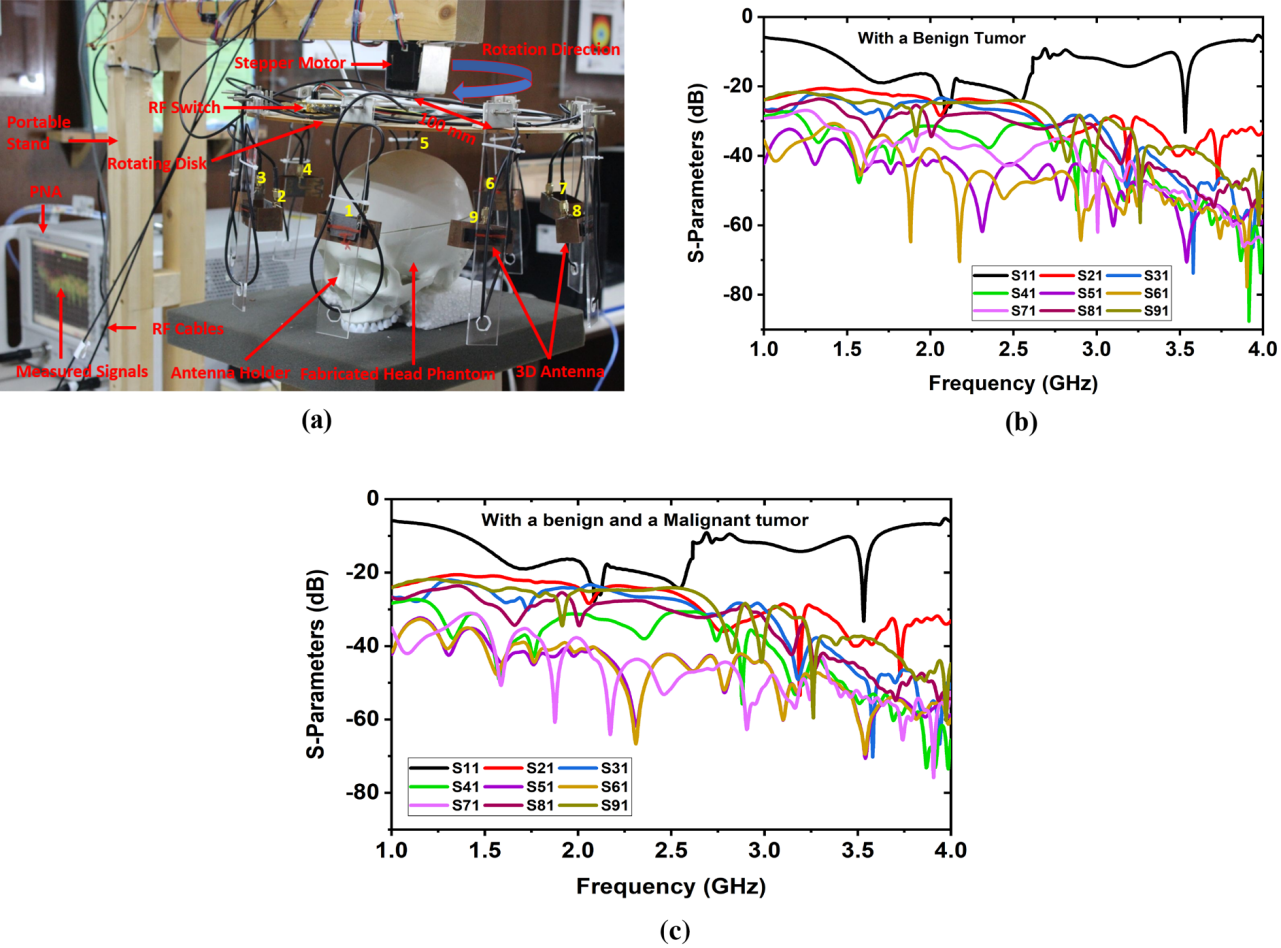
(**a**) Experimental MWHI system^19^, (**b**) S-parameters with a benign tumor, (**c**) S-parameters with a benign and a malignant tumor.

In the MWHI system, a wideband antenna is mandatory that must have the capability to operate a frequency band within the range of 1 GHz to 4 GHz with unidirectional radiation features^18,19,25,26^. A three-dimensional (3D) antenna was designed on cost-effective Rogers RT5880 substrate material having 1.575 mm thickness (Th), 2.20 dielectric constant, and 0.0009 loss tangent (δ). The improved dimension of the antenna is 60 × 25 × 16.575 mm^3^. The power network analyzer (PNA) was used to measure the antenna’s S-parameters(|S11|). Figure 1a illustrates the measured and simulated |S_11_| parameters of the antenna. It is observed from Fig. 1a, the measured operating frequency band of the antenna is 1.31 GHz to 3.65 GHz, while the simulated frequency band is 1.21 GHz to 3.67 GHz. It is noticeable that good agreement has been found in both measured and simulated outcomes except slightly shifting the resonances to the lower frequencies due to fabrication tolerance. After that, the antenna was verified with a Hugo head model by the CST 2019 simulation software and then measured with a fabricated tissue-mimicking head phantom to assess the performance steadiness of the fabricated antenna. Figure 1b depicts the measured and simulated |S_11_| parameters curve through the fabricated head phantom. It is seen from Fig. 1b, the |S_11_| of the antenna in head proximity keeps a good contract between the measured and simulated outcomes. In this research, a fabricated nine-antenna array was used in the developed MWHI framework^19^ for imaging, shown in Fig. 2a. Later, the system's performance was validated by the fabricated tissue-mimicking head phantom model, including benign and malignant tumors as brain abnormalities. The system has a portable stand with a circular-shaped rotating disk (0° to 360°) and a stepper motor. All nine antennas were attached with the separate nine antenna holders, and each antenna was set 40° apart from each other. The distance of each antenna from the center of the disk was 100 mm. The mounted array of the antenna rotates through the stepper motor from 0° to 360° around the fabricated tissue-mimicking head phantom. The S-parameters (scattering parameters) with a benign tumor are depicted in Fig. 2b, and with a benign and a malignant tumor is illustrated in Fig. 2c, when antenna 1 was excited, and others were received the scattered signals. The backscattered signals: S_11_, S_21_, S_31_……S_91_ were collected in each 7.2° degrees rotation. Thus, in-total 8 × 9 × 50 locations were scanned, and then the M-DMAS (modified-delay-multiply-and-sum) image reconstruction algorithm^19^ was applied to the collected backscattered signals to reconstruct the brain images of the head regions.

**Figure 3:**
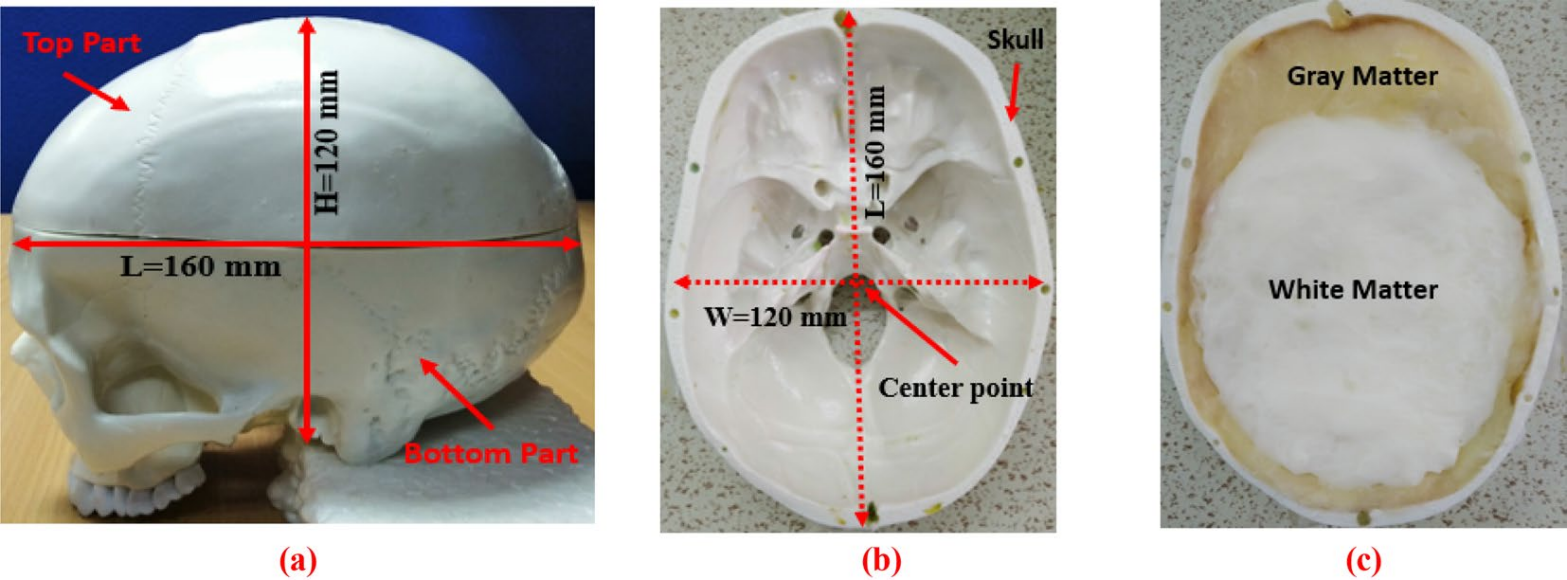
(**a**) Full 3D head skull model, (**b**) Internal structure of the blank model, (**c**) Fabricated tissues in the blank model.

**Figure 4:**
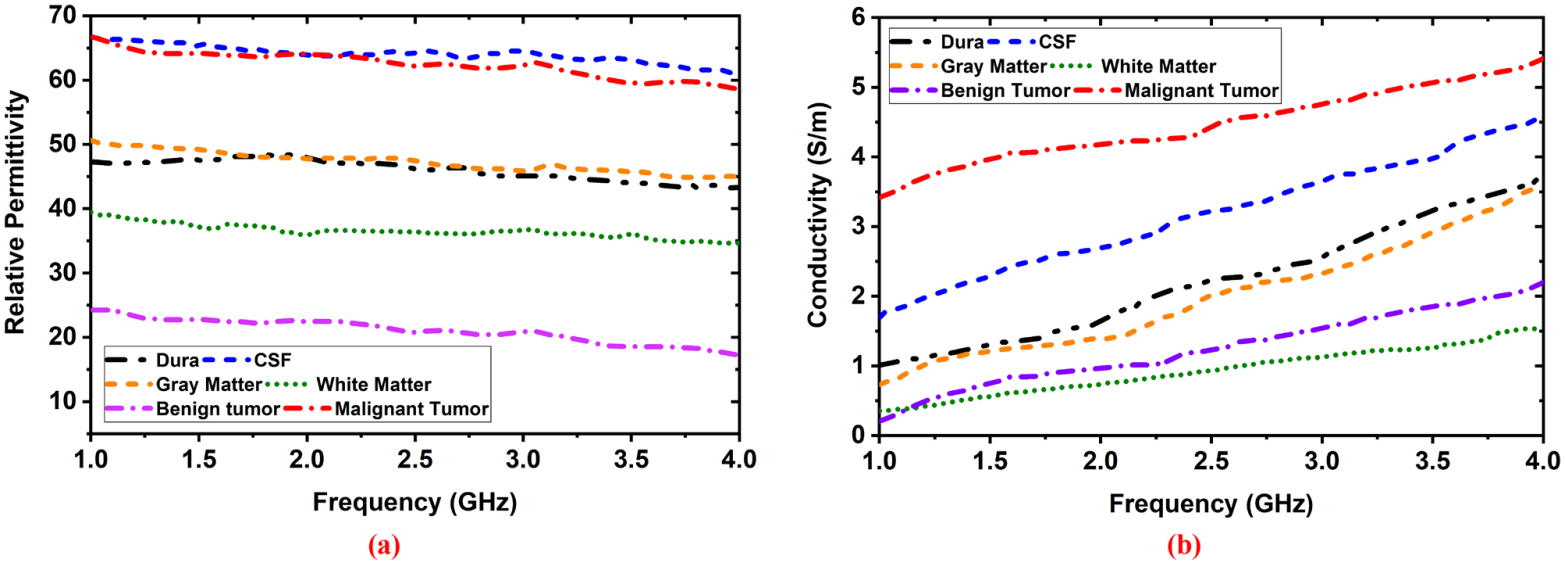
Measured dielectric properties of the fabricated tissues: (**a**) permittivity, (**b**) conductivity.

**Figure 5:**
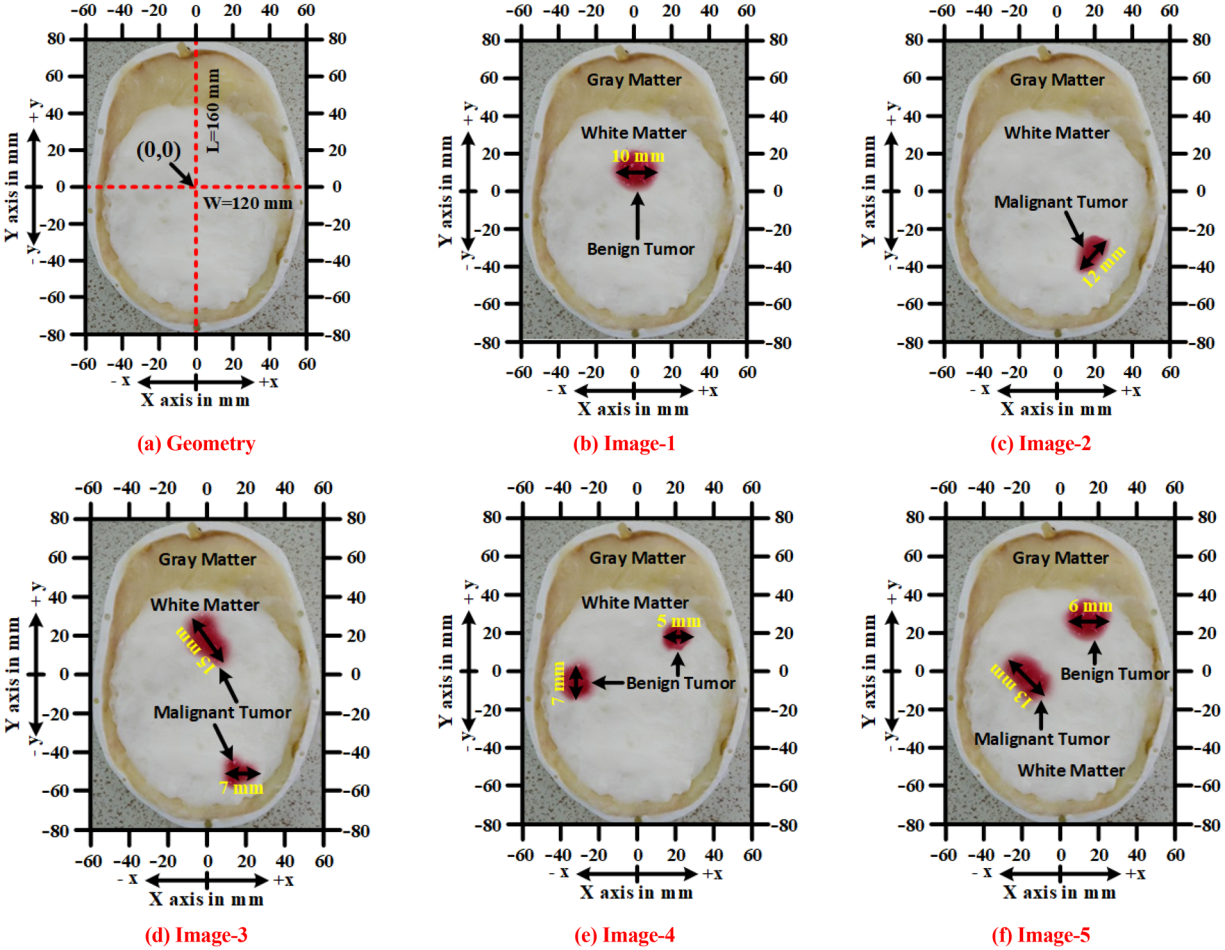
Fabricated head phantom’s geometry with tumor size and locations: (**a**) Overall geometry, (**b**) With a benign tumor, (**c**) With a malignant tumor, (**d**) With two malignant tumors, (**e**) With two benign tumors, (**f**) With one benign and one malignant tumor.

A human brain consists of four tissue layers: (i) Dura, (ii) CSF, (iii) Gray matter, and (iv) White matter. The brain tissues and tumors (i.e., benign and malignant ) are fabricated according to the presented formula in^50^ to verify the performance. The 3D skull model is depicted in Fig. 3a. The approximate length (L), width (W), and height (H) of the 3D skull head model are 160 mm,120 mm, and 120 mm respectively. The internal structure of the blank model is shown in Fig. 3b. Fabricated tissues are inserted into the blank model which is illustrated in Fig. 3c. The tumors' dielectric properties (i.e., permittivity and conductivity) are considered according to the presented value in^51^, which ensures the properties of the actual tumor. The fabricated brain tissues and tumors are measured by the dielectric probe kit KEYSIGHT 85070E and PNA. The measurement is performed within the frequency band of 1 GHz to 4 GHz. The measured dielectric properties of the tissues and tumors are presented in Fig. 4. The benign tumor is fabricated almost circular with a regular shape^52^. The considered diameters of the benign tumor are: D = 5 mm, 6 mm, 7 mm,10 mm, and 12 mm. On the other hand, the malignant tumor is fabricated as an elliptical and triangle irregular shape^52^. The considered approximate lengths of the malignant tumor are: L = 10 mm, 12 mm, 13 mm, 15 mm, and 16 mm. Then, the tumors are inserted into the different locations in terms of depth of the fabricated 3D head skull model to verify the system performance. The internal depth of the model is different at different locations. For better understanding, the fabricated model’s actual geometry with tumor diversity (i.e., tumor size, location, and shape) is presented in Fig. 5a–f. The tumor locations in terms of depth in the model are presented in Table 1. It is observed from Fig. 5b–f, the location can be identified by applying the cartesian coordinate method in the 2D image presentation. For instance, the benign tumor location in image-1 is 30 mm up from the phantom’s ground, and the cartesian coordinate is (0, 10) in mm. However, the detailed diversity of tumors in terms of four (4) aspects: (i) tumor size, (ii) location, (iii) morphology (shape), and (iv) heterogeneity (i.e., permittivity and conductivity) is presented in Table 1. Also, the various combinations of tumor size, location, and shape are used in phantoms to verify the performance, which is shown in Fig. 5b–f.

**Table 1:** The detailed diversity of the tumor(s) in terms of four aspects.

Phantom image	Tumor type	Tumor Size *D* for Benign,* L* for Malignant (mm)	Tumor location in terms of depth (from ground to up) (mm)	Center location of tumor(s) in Cartesian coordinate (x, y) (mm)	Tumor shape	Heterogeneity	
						Permittivity at 2 GHz	Conductivity at 2 GHz
Image-1	Benign	*D* = 10	30	(0, 10)	Circular with regular shape	22.65	0.965
Image-2	Malignant	*D* = 12	55	(20, − 30)	Elliptical with irregular shape	64.05	4.18
Image-3	Two Malignant	*D* = 7 and *L* = 15	30 and 55	(15, − 50) and (0, 15)	One is elliptical, and the other is a triangle with an irregular shape	64.05	4.18
Image-4	Two Benign	*D* = 5 and *L* = 7	40 and 35	(20, 15) and (30, − 7)	Circular with regular shape	22.65	0.965
Image-5	One benign and one malignant	*D* = 6 and *L* = 13	42 and 35	(12, 27) and (− 15, − 7)	One is circular with a regular shape, and the other is elliptical with an irregular shape	22.65 and 64.05	0.965 and 4.18

**Figure 6:**
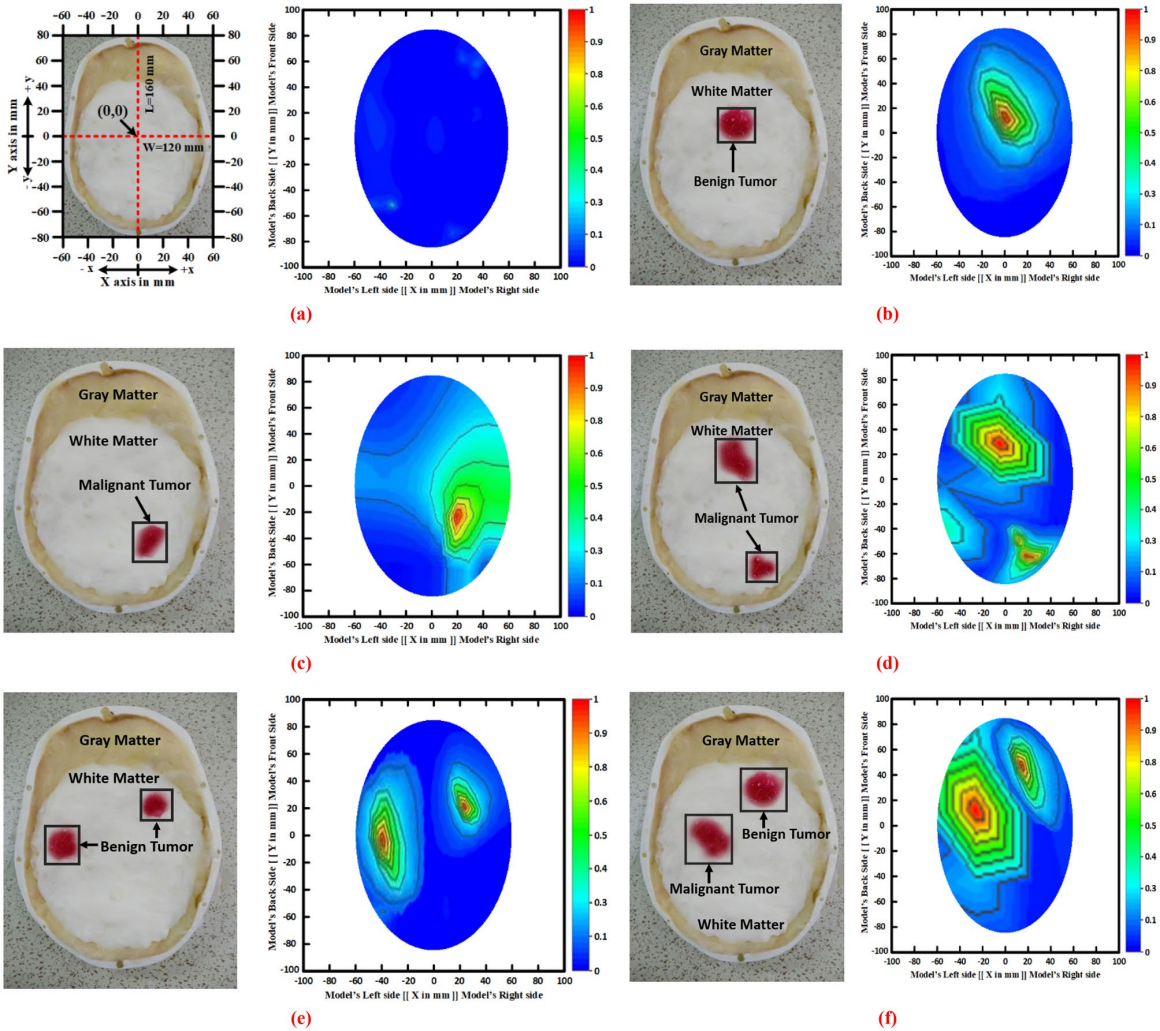
Fabricated head phantom model with brain abnormalities and corresponding reconstructed EM brain images (**a**) Without tumor image, (**b**) A single benign tumor image, (**c**) A single malignant tumor image, (**d**) Two malignant tumors image, (**e**) Two benign tumors image, (**f**) One benign and one malignant tumor image.

Figure 6 illustrates the fabricated phantom, different tumor shape, size with location, and reconstructed head region images. Moreover, for imaging purposes, a benign tumor, a malignant tumor, double benign tumors, double malignant tumors, and a benign and a malignant tumor as brain abnormalities in various locations were used to generate four-hundred reconstructed samples as a sample image dataset. After that, the image pre-processing and augmentation methods were applied to the sample dataset to create a training dataset as a final image dataset to train the deep neural network model. Furthermore, the YOLOv5 object detection models have been used to classify the tumors with objectness score rate automatically, and then identify the tumor position from the produced reconstructed microwave brain images. The YOLOv5 models have been implemented and executed by the Python high-level language with a PyTorch machine learning library.

## Background analysis of YOLOv5 model

### Background of Yolov5 algorithm

YOLO is the short term of "You look only once", and it is the deep learning-based object detection algorithm invented by Joseph Redmon et al.^53^. YOLOv5^54^ is also a deep learning-based object detection algorithm that improves the previous versions of YOLOv1 to YOLOv4, and it was recently released by the Ultralytics company^54^ on 27 May 2020. It is much faster and has a less computational architecture than the previous versions. The YOLOv5 model can balance detection accuracy and real-time performances. The YOLOv5 incorporates a one-stage deep convolutional neural network (DCNN) architecture that divides the images into a grid of cells. Straightly, each grid cell predicts a bounding box (BB) for object categorization. Every cell is liable to detect the target object as an item and expects BB with the confidence score (CS)^53^. In YOLOv5, the CS replicates whether a target object exists in a cell or not and predicts the object accurately. The CS is calculated by using the following Formula (1):

1 $$ CS = P_{r} (T_{object} ) \times IoU(GT,PB) $$

where $P_{r} (T_{object} )$ is the prediction of the target object ($T_{object}$) and $P_{r} (T_{object} ) \in \{ 0,\,\,1\}$, intersection over union ($IoU$) calculated by between the *GT* and *PB.* GT means Ground Truth, and PB means predicted box. It is noticeable that, if no target object presents in the grid cell, the CS should be one (1); otherwise, the CS should be the IoU between the *GT* and *PB.* In addition, each cell predicts C_L_-class probabilities for identifying the objects. However, the predicted output tensor (POT) is expressed by using the Eq. (2) as follows:

2 $$ POT = BB \times (5 + C_{L} ) $$

Here, *BB* is the bounding box for each grid prediction, and it is considered as 3, the “5” denotes every prediction box's four coordinates [x, y, w, h], and one object confidence score (CS). In this instance, [x, y] denotes the middle position’s coordinates of the box, and [w, h] indicates the box's width and height, respectively. C_L_ represents the class probabilities. Hence an object can exist in numerous classes; thus, a single 1 × 1 convolutional layer, sigmoid, and Leaky ReLu activation functions are used in YOLOv5. In addition, the activation function Leaky ReLu is applied in intermediate or hidden layers, and the sigmoid function is involved in the ending prediction layer. The CS of each class is projected by the Leaky ReLu, sigmoid activation function, and a threshold value responsible for identifying the target object. In YOLOv5, the Binary Cross-Entropy with Logistic Loss (BCELL) function from the PyTorch library is used for calculating the loss of class probability and target object scores^55^. The estimated loss integrates a sigmoid layer and a BCELoss function in a single class. The unreduced and multilevel classification loss functions in BCELL are expressed by the following equations^55^:

3 $$ L(x_{i} ,y{}_{i}) = L = \{ \ell_{1} ,\ell_{2} ,......\ell_{N} \}^{T} $$

4 $$ \ell_{ns} = - w_{ns} [y_{ns} .\log \sigma (x_{ns} ) + \,(1 + y_{ns} ).\log (1 - \sigma (x_{ns} ))] $$

5 $$ L_{c} (x_{i} ,y_{i} ) = L_{c} = \{ \ell_{1,c} ,........\ell_{N,c} \}^{T} $$

6 $$ \ell_{ns,c} = - w_{ns,c} [p_{c} y_{ns,c} .\log \sigma (x_{ns,c} ) + (1 - y_{ns,c} ).\log (1 - \sigma (x_{ns,c} ))] $$

Here, *N* denotes the batch size, *c* represents the class number, (x_i_, y_i_) is the coordinate value of predicted boxes, the offsets of the center locations are presented by $\sigma (x_{ns} )$ and $\sigma (x_{ns,c} )$, *ns* is the number of samples in the batch, $p_{c}$ denotes the weight of class *c*. If c = 1, it would be a single-label binary classification, and if *c* > 1, it would be a multi-label binary classification.

### Non-maximum suppression (NMS) technique

An image contains multiple target objects, and the objects might be of different shapes and sizes. So, the target objects might be captured perfectly with a single bounding box. The YOLOv5 model creates more than one overlapping bounding box (BB) in a single image to detect target objects but needs to show only a single bounding box for each object in an image. Thus, we need a method that eliminates overlapping bounding boxes. Therefore, the Non-Maximum Suppression technique is applied to eliminate the overlapping problem, selecting a single BB out of more than one overlapping BB to identify the objects in an image. The NMS method removes the redundant identifications and determines the best match for ending identification. The NMS technique is presented in Algorithm 1. Besides, the YOLOv5 decides on GIoU loss^56^ as the BB regression loss function to solve the inaccurate calculation of non-overlapping BB and defined as following equation^56^:

7 $$ L_{GIoU} = 1 - IoU + \frac{{\left| {C_{B} - PB \cup B^{GT} } \right|}}{{\left| {C_{B} } \right|}} $$

where $B^{GT}$ represents the GroundTruth box, $PB$ represents the predicted box, *C*_*B*_ represents the smallest box covering $PB$,$B^{GT}$, and $IoU = \frac{{PB \cap B^{GT} }}{{PB \cup B^{GT} }}$.
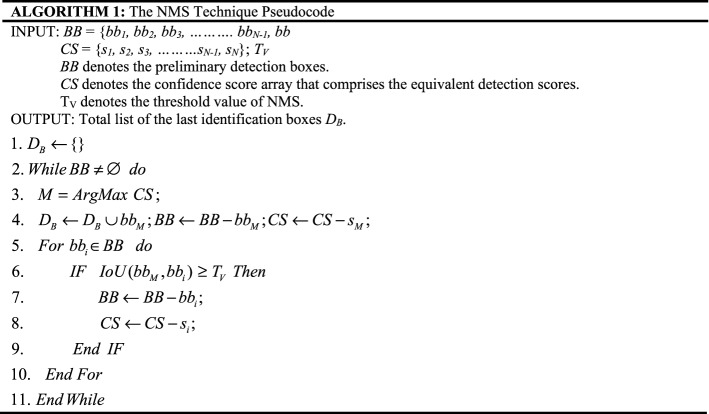


## Classification and detection methodology and materials

**Figure 7:**
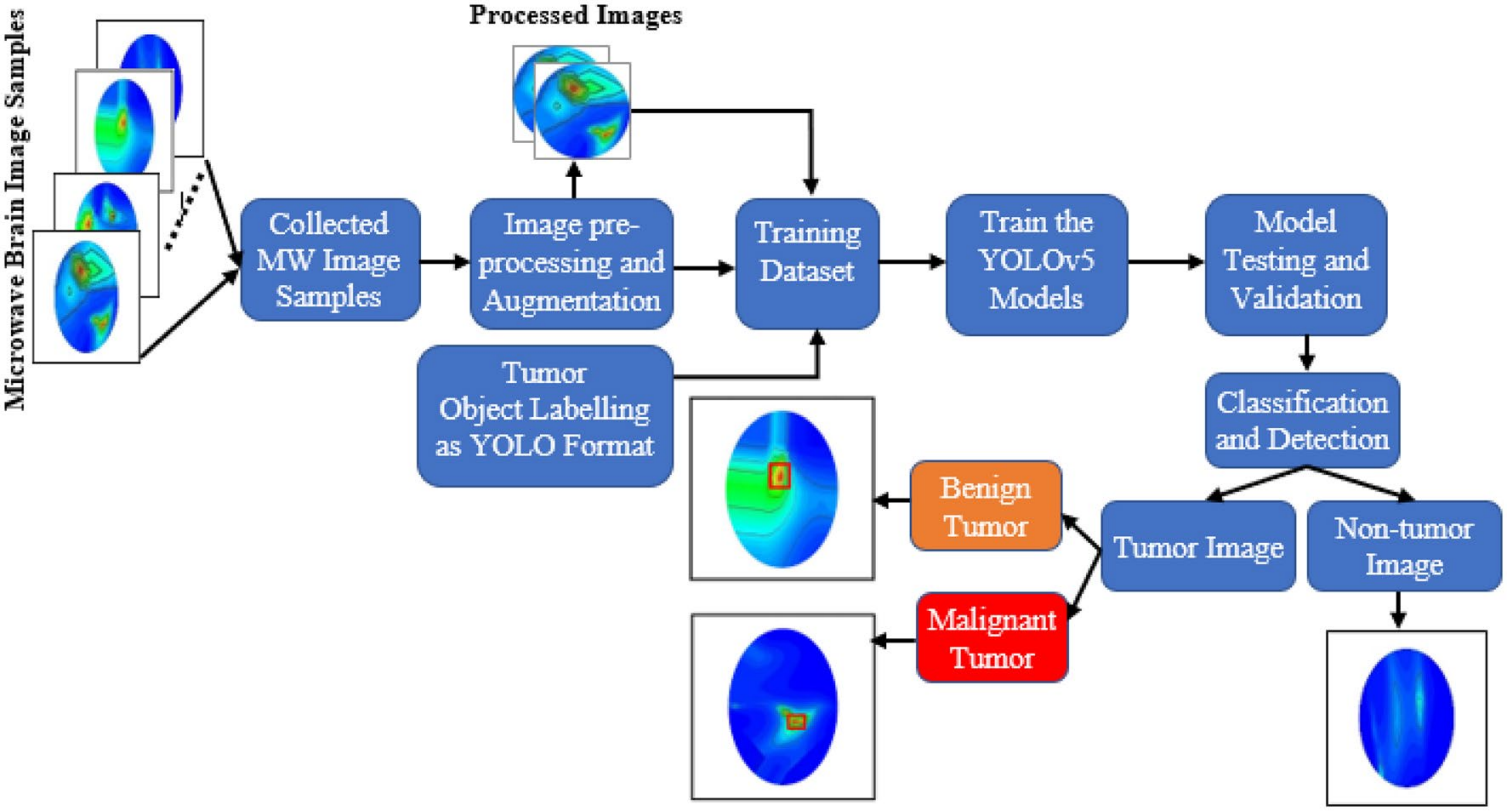
Overall flow chart of the proposed methodology.

This section discusses the overall research methodology, dataset explanation, image pre-processing, augmentation techniques, YOLOv5 architecture with classification analysis, and detection procedure. The complete methodology of the classification and detection flow chart is portrayed in Fig. 7. This study utilized the reconstructed microwave (RMW) brain images collected from the experimental MWHI system. The processed images and their corresponding labeling of tumor objects in YOLO format are being used to train the YOLOv5 models. As mentioned earlier, the work used mainly different types of images such as (i) Non-tumor image (ii) a single benign tumor image, (iii) a single malignant tumor image, (iv) double benign tumors image, (v) double malignant tumors image, and (vi) a single benign and a single malignant tumor image. The work also explored different categories of reconstructed microwave head images to investigate the performance of the YOLOv5 models that can automatically detect the tumors with location and classify the tumor(s) into two classes (i.e., benign and malignant).

### Dataset explanation

**Figure 8:**
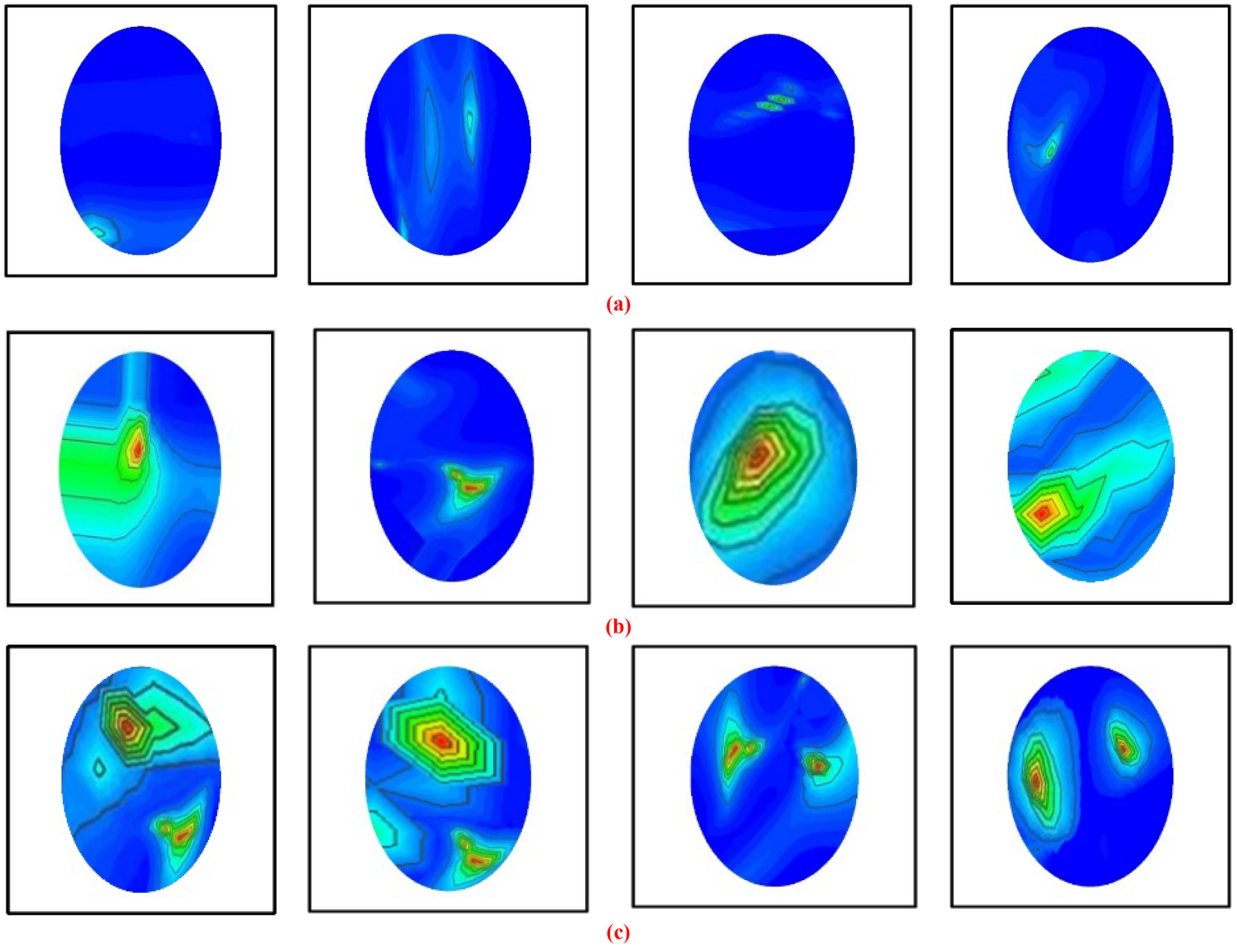
Randomly selected RMW brain image samples: (**a**) Non-tumor image, (**b**) With a benign and a malignant tumor image, (**c**) With double benign and double malignant tumor(s) image.

In this study, the original four-hundred RMW brain image samples were initially collected from the implemented MWHI system to create a sample dataset. The sample dataset contains one hundred samples for non-tumor images, seventy-five samples for every single benign tumor and single malignant tumor image, fifty image samples for each double benign tumor(s), double malignant tumor(s), and single benign and single malignant tumor. Every image size is 640 × 640 pixels, which is the input requirement for the proposed models. After that, the image pre-processing and augmentation techniques are applied to the sample dataset to generate a training dataset. The training dataset consists of 4400 images, where 80% used for training and 20% used to test the YOLOv5 model. Also, out of 80% of images, 20% were used for validation to avoid overfitting. For instance, the RMW brain image samples are illustrated in Fig. 8.

### Image pre-processing and augmentation techniques

**Figure 9:**
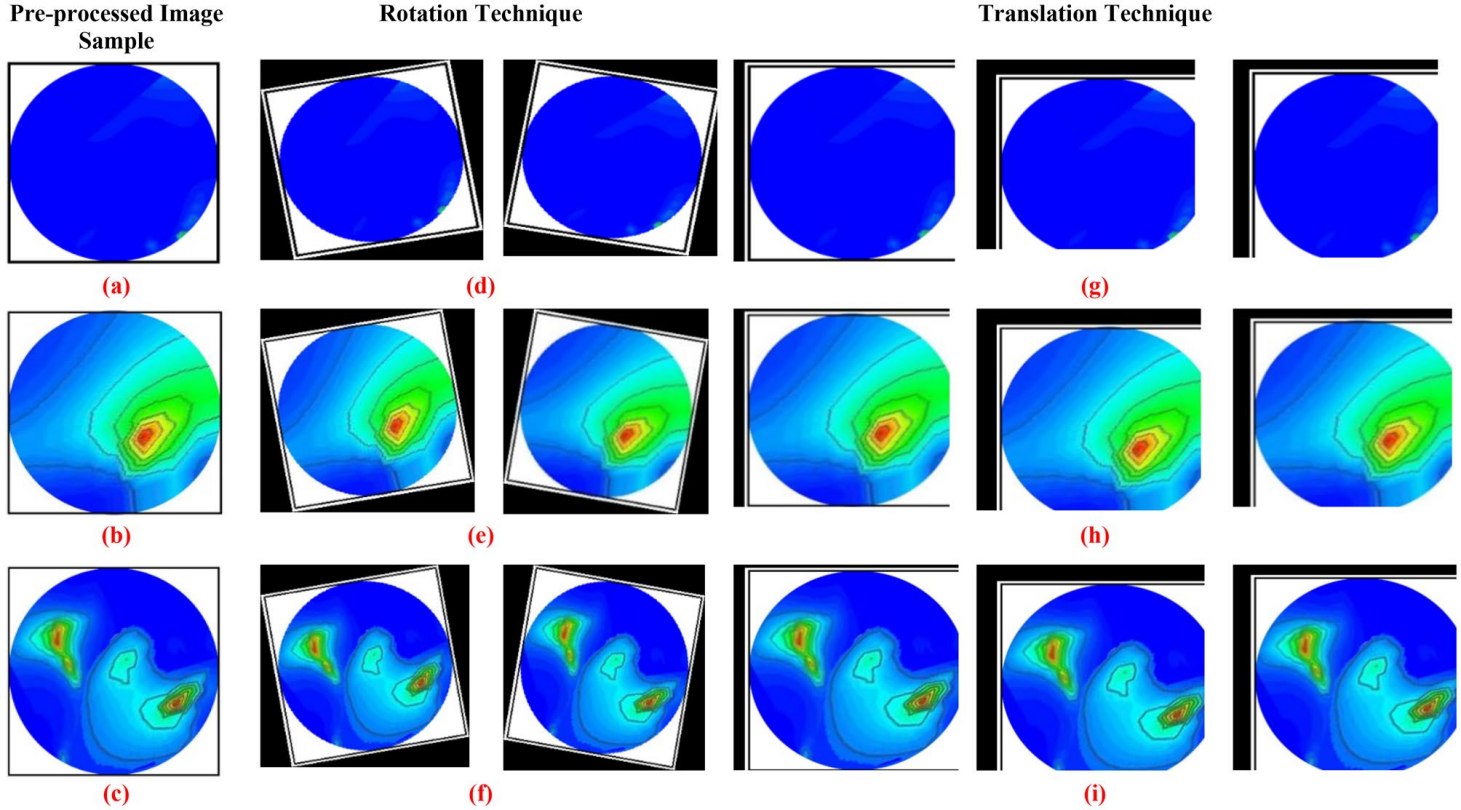
Randomly selected images from the augmented training set: (**a**–**c**) Non-tumor, single benign, and one benign and one malignant tumor images, (**d**–**f**) Images after rotation by 30 degrees anticlockwise and clockwise for non-tumor, single benign, and one benign and one malignant tumor, (**g**–**i**) Images after 3% horizontal, 5% vertical and horizontal, 5% horizontal and 3% vertical translation for non-tumor, single benign, and one benign and one malignant tumor.

The image pre-processing technique is the first phase of a deep learning model due to its input image size requirements. For example, the deep learning-based YOLOv5 object detection model requires input size. Thus, the images are pre-processed before training the network model. Initially, every image's head region area is cropped, then the resize and normalization techniques are applied to the sample dataset. After that, the images are resized to 640 × 640 pixels to satisfy the input requirements of the YOLOv5 model. It is noticeable that the YOLOv5 model requires a large dataset to be trained efficiently to classify and detect the target tumor with locations in the RMW brain images. Since we have a four hundred image sample dataset, which is not suitable for training the model; thus, image augmentation techniques are applied to the sample dataset to generate a training dataset for effectively training the model. The image augmentation can enhance the classification and detection accurateness of the model by augmenting the existing image dataset rather than the collection of new samples. In addition, image augmentation can remarkably enhance the variety of data available for the training model and create a rich dataset from the insignificant image sample dataset for image categorization. This study utilizes eight different image augmentation strategies: rotation, scaling, translating, horizontal flipping, vertical flipping, width shifting, height shifting, and zooming to generate a training dataset. The rotation operation of images is accomplished by rotating anticlockwise and clockwise directions with an angle between 3° to 30°. As a result, the tumor objects are shifted at different positions in the images. Scaling is the reduction or magnification of the image frame size. So, using the scaling method, 2% to 15% image magnifications are used for augmentation. In addition, the translation technique translates the images vertically and horizontally by 3% to 10%. Moreover, 5% to 15% width and height shifting are applied to the images for shifting the tumor objects. Also, the probability factor of 0.2 to 0.5 is applied for vertical and horizontal flipping purposes. Thus, the tumor objects have changed the position in the images. However, taking a single sample image from the four hundred samples, then applying all augmentation strategies separately to produce eleven augmented images at a time programmatically. As a result, obtained augmented number of images as a training dataset is 11 × 400 = 4400. The detailed dataset description is presented in Table 2. For instance, some augmented images are illustrated in Fig. 9.

**Table 2:** Dataset description for training, testing, and validation.

Dataset	Number of original image samples	Type of images	Number of images	Augmented images	Training dataset		
					Number of training images (80%)	Number of validation images (20%)	Number of testing images (20%)
Reconstructed microwave (RMW) brain images	400	Non-tumor	100	1100	3520	704	880
		Single benign tumor	75	825			
		Single malignant tumor	75	825			
		Double benign tumor	50	550			
		Double malignant tumor	50	550			
		Single benign and single malignant tumor	50	550			
Total			400	4400	3520	704	880

### Target object labelling

**Figure 10:**
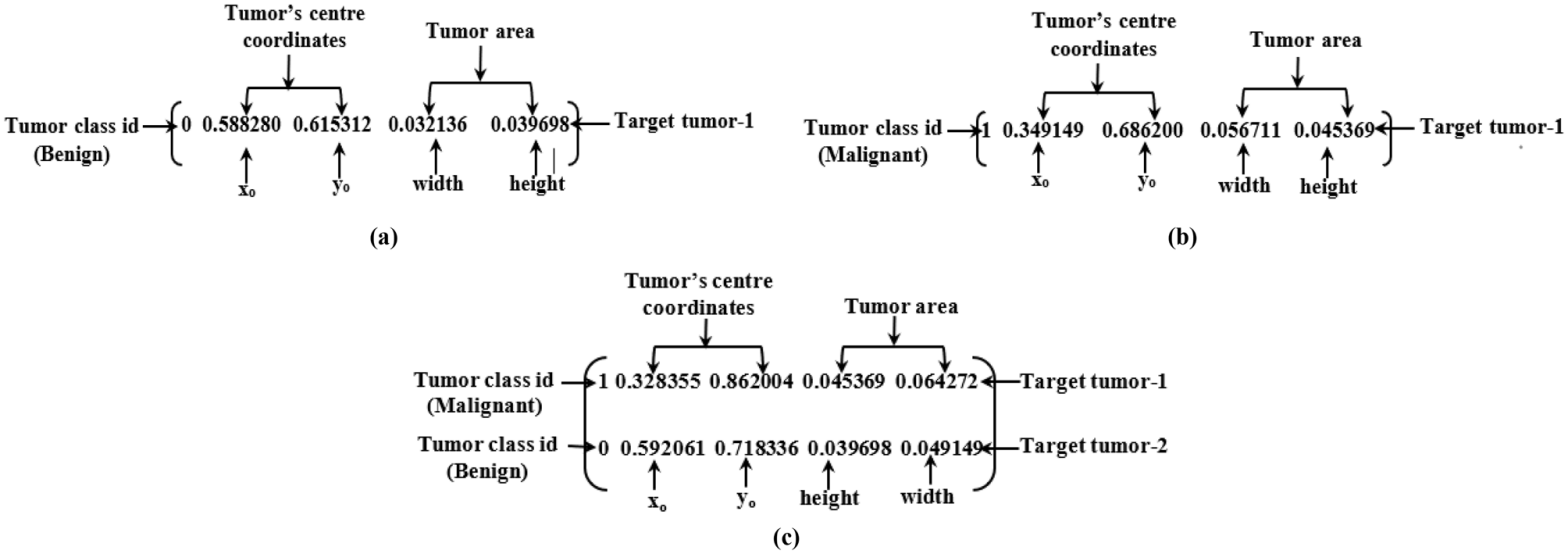
YOLO format file context: (**a**) For benign tumor as a single object, (**b**) For malignant tumor as a single object, (**c**) For malignant and benign tumor as two tumor objects.

Labeling tumor objects in the image dataset is necessary for training the YOLOv5 models. The YOLOv5 object detection model is pre-trained on Microsoft Common Objects in Context (MSCOCO) dataset^54^. The dataset consists of 80 predefined classes, and the class names are stored in "yaml" file. But, our tumor object class names (i.e., benign and malignant) and dataset are not included in the predefined dataset of YOLOv5 model. Thus, we created a separate "yaml" class file and a YOLO tumor object labeling format file in this study. The "yaml" file consists of two classes: one is benign tumor class, and the other is malignant tumor class. On the other hand, the annotation process is applied to every image of the final image dataset to generate a tumor object labeling file as a YOLO format file. The YOLO format file is a text file with a similar name of each image that contains the tumor class id, tumor object's center coordinates, object's height, and object's width (i.e., tumor labeling area). For tumor object labeling purposes, a graphical image annotation tool LabelImg is used to create an annotation file for every image. The tool uniformly labels each tumor object from the training and validation dataset. Finally, the training dataset and corresponding YOLO format files are used to train the network model. For instance, after annotation, the YOLO format file context is presented in the following Fig. 10. The contents of the YOLO format file with a single benign tumor, single malignant tumor, and malignant and benign tumor objects are depicted in Fig. 10. A txt file has three components for every target tumor object: tumor class id, center coordinates of the tumor bounding box (x_o_, y_o_), and tumor area (width, height). The id "0" represents the benign tumor class, and "1" represents the malignant tumor class. The YOLO format file of benign tumor, malignant tumor, malignant and benign tumor labeling information are portrayed in Fig. 10a–c.

### Tumor-Yolov5 architecture model

**Figure 11:**
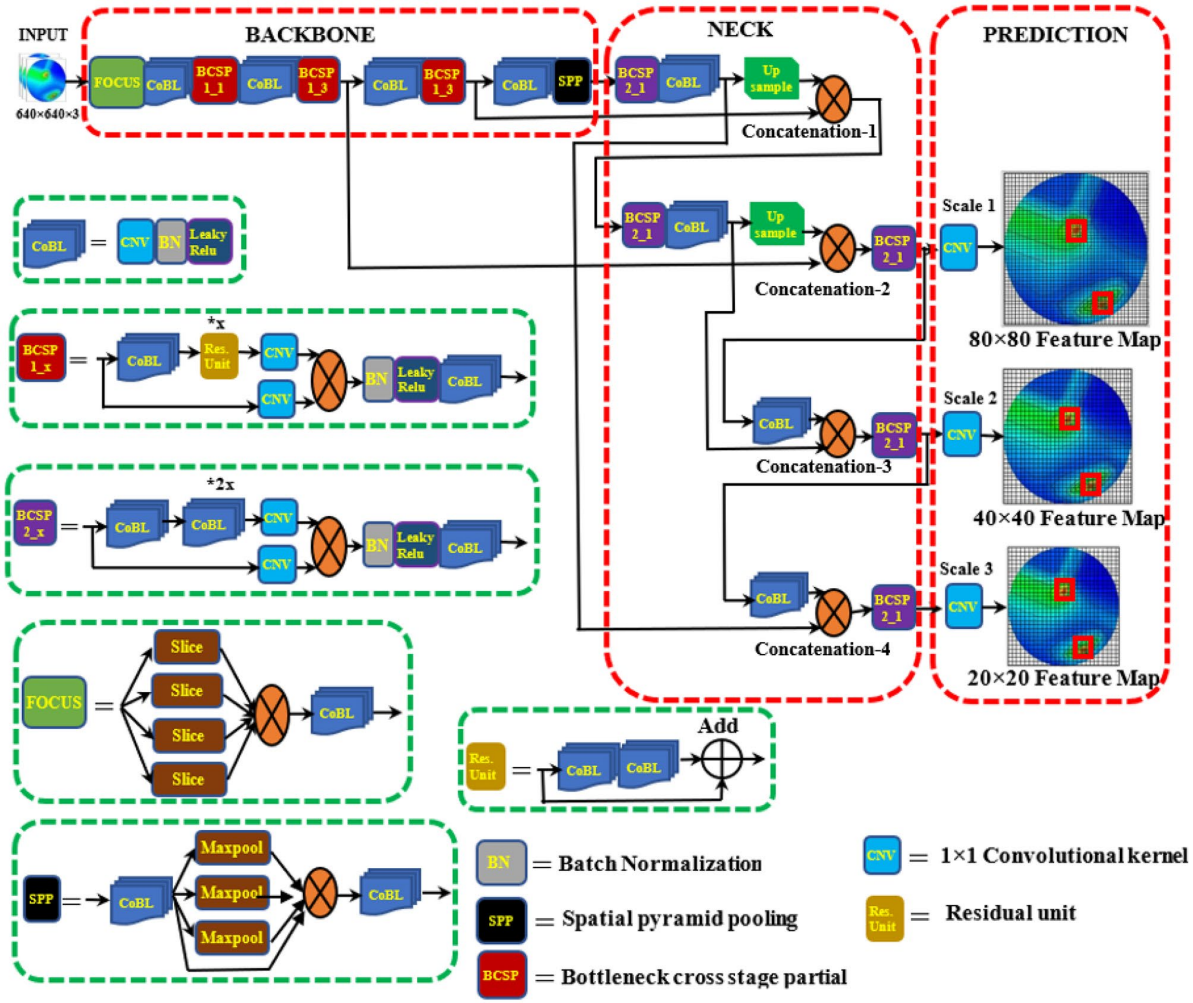
YOLOv5 architecture model.

The YOLOv5 architectural model is portrayed in Fig. 11. The architecture is divided into three parts: (i) Backbone, (ii) Neck, and (iii) Prediction. *Firstly*, the Bottleneck Cross Stage Partial Darknet (BCSPD) is the main backbone of the YOLOv5 framework. The input image dimension of YOLOv5 is 640 × 640 × 3 pixels. After feeding the images into the model, the output could be any combination of desired classes. The input image goes through the FOCUS module in the backbone. The FOCUS module slices the input image into four small ones and then concatenates them together for convolutional operation. The 640 × 640 × 3 pixels image is divided into four small images with the dimension of 320 × 320 × 3, then that are concatenated into a 320 × 320 × 12 pixels feature map. Thereafter, 32 convolutional kernels operation, it becomes a 320 × 320 × 32 feature map. The CoBL module of the model is a fundamental convolutional module that epitomizes Conv2D + Batch Normalization (BN) + Leaky ReLu activation function. The BCSP mainly performs the feature extraction on the feature map, extracting major information from the feeding images. It solves the repeated gradient information duplication in CNNs and integrates the gradient changes into the feature map, thus reducing the input parameters and size of the model. The BCSP consists of a residual unit and two 1 × 1 Conv2D kernels. A residual unit contains the two CoBL modules and adder. The adder adds the features of the previous CoBL module and two CoBL modules output then sends local features to the one 1 × 1 Conv2D layer. The four models with different input parameters can be attained, namely YOLOv5s, YOLOv5m, YOLOv5l, and YOLOv5x by adjusting the width(w) and depth(d) of the BCSP module. Besides, the Spatial Pyramid Pooling (SPP) module integrates with the BCSP module in the backbone. The SPP module increases the receptive field of the network and acquires features of various scales. *Secondly,* YOLOv5 integrates the Path Aggregation Network (PANet) in the neck to enhance the information flow^57^. The PANet is based on the Feature Pyramid Network (FPN) structure that conveys robust semantic features from top to bottom. Also, FPN layers convey strong positioning features from bottom to top. In addition, PANet improves the propagation of low-level features and the utilization of accurate localization signals at the bottom layers. Thus, the location accuracy of the target object is enhanced. *Thirdly*, the prediction layer is also called the detection or YOLO layer, which generates three different feature maps. The feature map is used to attain multi-scale prediction^58^. As a result, the model can classify and detect small, medium, and large objects at the prediction layer, including a bounding box. The prediction process summary of the YOLOv5 model is discussed below:

#### Phase 1

Initially, the 640 × 640 size of images is applied as an input to the backbone. After that, the images are sliced by the FOCUS module. After executing many convolutions set of operations and two times BCSP1 operations, the feature map goes to the second concatenation layer. On the other hand, after the one-time execution of BCSP1, two times of BCSP2, convolutions set of operation, and two times of upsampling, the feature map goes to the second concatenation layer. The second concatenation layer concatenates both of them. After execution of BCSP2 layers and 1 × 1 convolution operation, the 80 × 80 sized feature map (i.e., scale 1) is obtained.

#### Phase 2

In the second step, the 80 × 80 sized feature map from phase 1 is processed by one 3 × 3 convolutional kernel and goes to the third concatenation layer. Besides, the earlier second upsampling feature map is executed by one 1 × 1 convolutional kernel and goes to the third concatenation layer. Then, the third concatenation layer concatenates both of the layers. After finalizing the BCSP2 layer and one 1 × 1 convolution operation, the 40 × 40 sized feature map (i.e., scale 2) is attained.

#### Phase 3

In the third phase, the 40 × 40 sized feature map from phase 2 is processed by one 3 × 3 convolutional kernel and goes to the fourth concatenation layer. Besides, the earlier upsampling feature map is executed by one 1 × 1 convolutional kernel and goes to the fourth concatenation layer. Then, the fourth concatenation layer concatenates both of the layers. Later, by performing the BCSP2 and 1 × 1 convolution operation, the 20 × 20 sized feature map (i.e., scale 3) is obtained.

#### Phase 4

Finally, feature maps in different sizes (i.e., 80 × 80, 40 × 40, 20 × 20) are responsible for identifying the different sized tumor objects with a regression bounding box (BB). Henceforth, at every location, predicts three (03) regression bounding boxes for each feature map, thus creating the 3 × (80 × 80, 40 × 40, 20 × 20) = 25,200 regression bounding boxes. Finally, the model predicts the location of the target tumor(s) with a bounding box with an objectness score.

### Classification and detection process

The YOLOv5 model is trained on MSCOCO dataset that comprises eighty (80) predefined object classes^54^. According to Eq. (2), the predicted output tensor (POT) dimension is 3 × (5 + 80) = 255. Here, "3" denotes the three bounding boxes (BB) for each grid cell prediction, "5" denotes the coordinates (x_o,_ y_o_, w, h) of each prediction box and confidence score (CS), and "80" represents the predefined object class (C_L_). In our study, we have two objects in the RMW brain images, one is a benign tumor, and the other is a malignant tumor. Thus, we need to modify the classifier of the YOLOv5 model. So, according to Eq. (2), the predicted output tensor (POT) dimension is 3 × (5 + 2) = 21 in our case. Due to the modification of the model, we get some significant benefits: (i) reduce the number of network parameters, (ii) reduce the computational overhead, (iii) enhance the detection accuracy, and (iv) increase the training and testing speed. After training the network, the testing, classification, and detection phases are started. The network model evaluates the testing dataset. Finally, the model classifies the tumor into benign and malignant classes. Also, it shows the location of the tumor(s) with a bounding box, including the objectness score.

## Details explanation of the training experiments

The YOLOv5 model was implemented and executed on the anaconda distribution platform using the PyTorch machine learning library with Python 3.7 version. The experiments have been carried out on a 64-bit Windows 10 operating system with 128 GB RAM and 64-bit Intel® Xeon®W-2016 @ of 3.30 GHz CPU. In addition, an 11 GB NVIDIA GeForce GTX 1082Ti GPU has been used to enhance the network training performances. In this study, three versions of the YOLOv5 model: YOLOv5s, YOLOv5m, and YOLOv5l have been carried out to evaluate the tumor classification and detection performances. In the experiment, the training dataset and corresponding annotation tumor class files have been utilized for training all models. The training dataset consists of 4400 images and their related labeling files. Later, 80% of the total images were utilized for training and 20% for testing. From the 80% training dataset, 20% has been utilized for validation to avoid overfitting. After that, a modified "ymal" class file and pre-trained learning weights for all models have been employed during the training period. The models have been trained for 200 epochs with an initial learning rate of 0.01, and the batch size is 16. The stochastic gradient descent (SGD) optimizer has been applied to optimize the model outputs. The detailed training hyper-parameters for the models are presented in Table 3.

**Table 3:** Training hyperparameters of YOLv5 models.

Name of the parameter	Value	Name of the parameter	Value
Initial learning rate	0.01	Weight decay	0.0005
Learning factor	0.2	Warmup epochs	3.0
Momentum	0.937	Warmup momentum	0.8
Optimizer	SGD	Warmup initial bias learning rate	0.1
IoU training threshold	0.20	Box loss gain	0.05
Anchors	3.0	Anchor multiple thresholds	4.0

### Performance evaluation metrics

The tumor classification with detection performance of the YOLOv5 network model is evaluated by the five-evaluation metrics such as (i) Precision (P), (ii) Sensitivity/Recall (R), (iii) Specificity (S), (iv) F1-score (Fs), and (v) Mean Average Precision (mAP). Precision represents the ability of a model that can detect only the relative tumor objects. On the other hand, recall/sensitivity means the ability of a model to find out all the relevant cases. The evaluation matrices are calculated by using the following equations:

8 $$ Precision\,(p) = \frac{{N_{TP} }}{{(N_{TP} + N_{FP} )}} $$

9 $$ Sensitivity/Recall\,(R) = \frac{{N_{TP} }}{{(N_{TP} + N_{FN} )}} $$

10 $$ Specificity\,(S) = \frac{{N_{TN} }}{{N_{TN} + N_{FP} }} $$

11 $$ 1 - Specificity(S) = \frac{{N_{FP} }}{{N{}_{TN} + N_{FP} }} $$

12 $$ F_{s} = \frac{{(2 \times N_{TP} )}}{{(2 \times N_{TP} + N_{FN} + N_{FP} )}} $$

13 $$ mAP = \sum\limits_{i = 1}^{N} {P(i) \times \Delta R(i)} $$

where $N_{TP}$ represents the number of tumor images was identified as tumors, $N_{FP}$ represents the number of images was identified as tumor errors, $N_{FN}$ represents the number of images that were missed identified as tumors,$N{}_{TN}$ represents the number of images that were missed classified as the tumor, $P(i)$ is the precision, and $\Delta R(i)$ is the change in recall from the ith detection.

## Result and discussion

This section discusses the experimental investigation of the training and testing performances, tumor classification, and tumor detection in RMW brain images by applying YOLOv5 models. The training dataset consists of 4400 images, including benign and malignant tumor(s), where 80% of the total images were utilized for training and 20% for testing the models. In further, out of 80% training dataset, 20% of images were used for validation to avoid overfitting.

### Training and validation performance analysis

**Figure 12:**
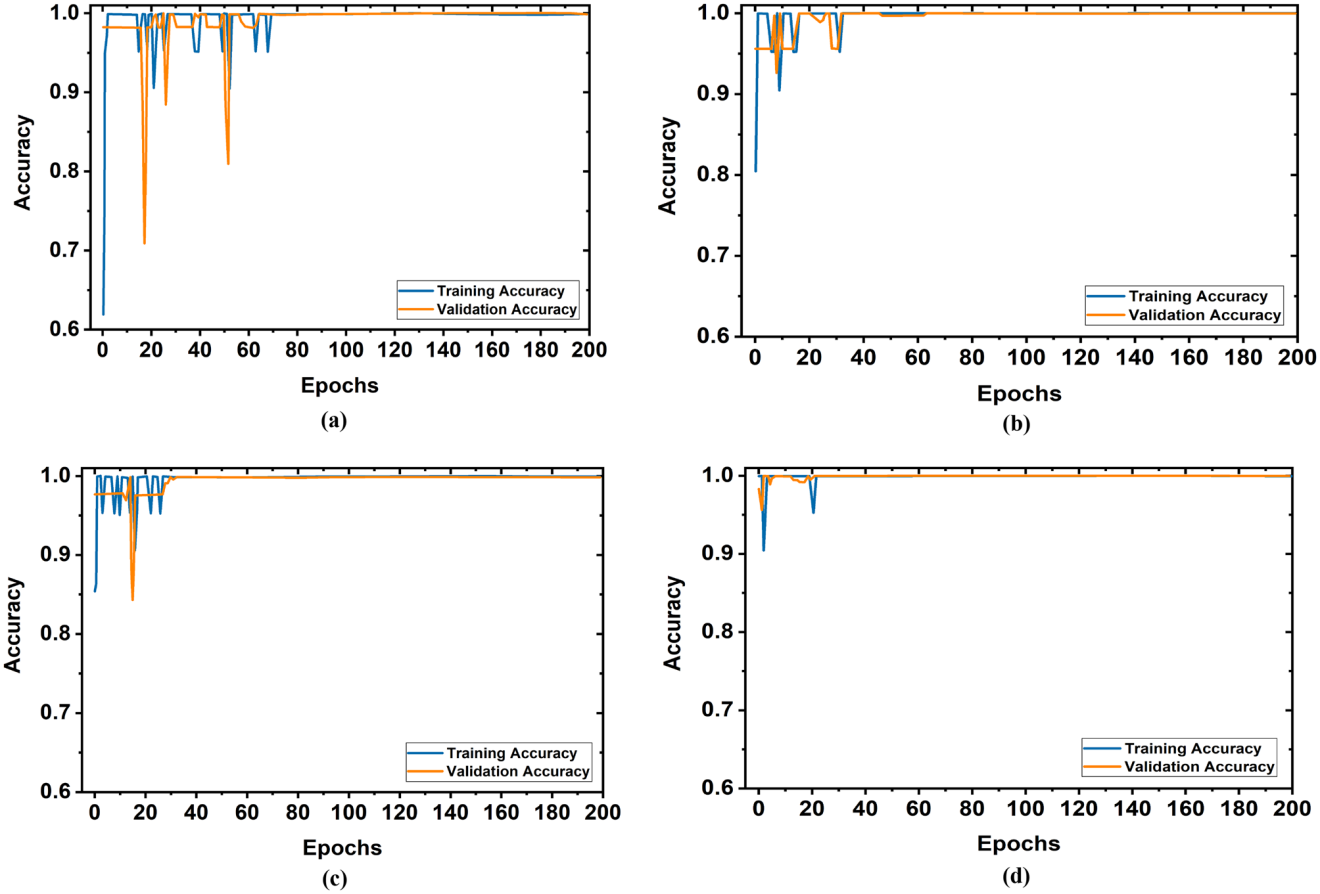
Training vs validation accuracy of YOLOv5l model for different training dataset: (**a**) 1000 images, (**b**) 2000 images, (**c**) 3000 images, (**d**) 3520(80%) images.

**Figure 13:**
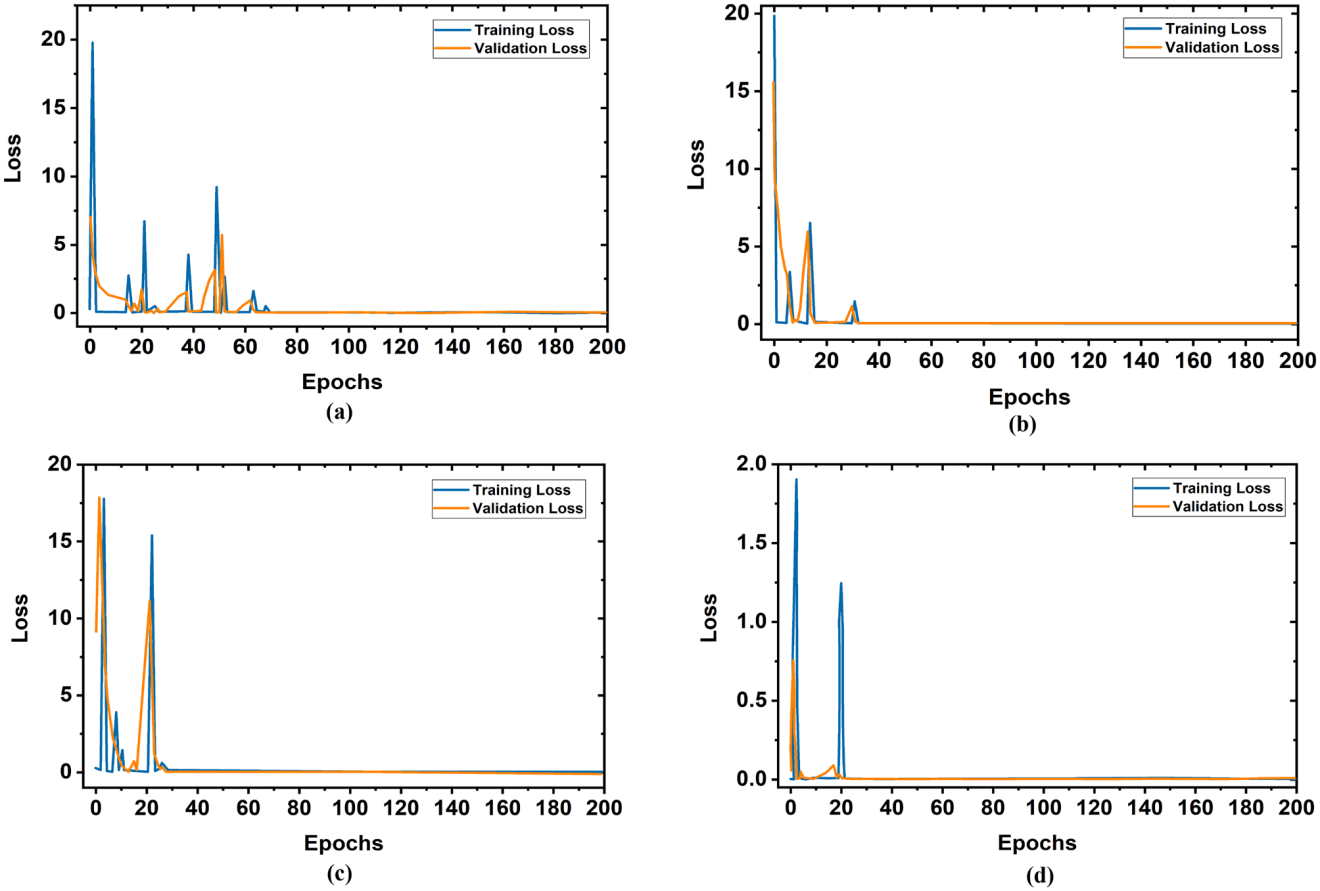
Training vs validation losses of YOLOv5l model for different training datasets: (**a**) 1000 images, (**b**) 2000 images, (**c**) 3000 images, (**d**) 3520(80%) images.

We have implemented three versions of the YOLOv5 model: YOLOv5s, YOLOv5m, and YOLOv5l to investigate the training and validation accuracy and loss performances by the different training datasets. It is seen that the YOLOv5l model showed the best performances. For simplicity, the training and validation accuracy graphs of the YOLOv5l model by applying different training datasets are illustrated in Fig. 12. It is observed from Fig. 12 when 1000 images were used as a training dataset then, initially, the training and validation accuracy was very low. But after 78 epochs, the model has achieved 99.99% accuracy in both cases. Also, when training dataset size is increased gradually as 2000, 3000, and 3520 (80%) to train the model, the training and validation accuracy is increased up to 99.99%, after 60, 35, and 22 epochs, respectively. In contrast, the training vs. validation loss graphs of the YOLOv5l model is illustrated in Fig. 13. It is observed from Fig. 12 when 1000 images were used as a training dataset, then initially, the training and validation loss is very high. But after 72 epochs, the loss of the model is approximately 0. Also, when training dataset size increases as 2000, 3000, and 3520 (80%) to train the model, the training and validation losses are decreased. The loss of the model is approximately 0 after 22 epochs. The overall average accuracy and loss outcomes of the YOLOv5l model are shown in Table 4.

**Table 4:** Overall outcomes of training versus validation accuracy and loss.

Training dataset	Average training accuracy (%)	Average validation accuracy (%)	Average training loss (%)	Average validation loss (%)
1000	96.02	94.98	2.18	3.08
2000	96.95	96.45	2.05	2.29
3000	97.77	97.34	1.02	1.01
3520 (80%)	99.94	99.68	0.0416	0.0918

### Investigation of tumor object classification and prediction

#### Classification and prediction parametric analysis

**Figure 14:**
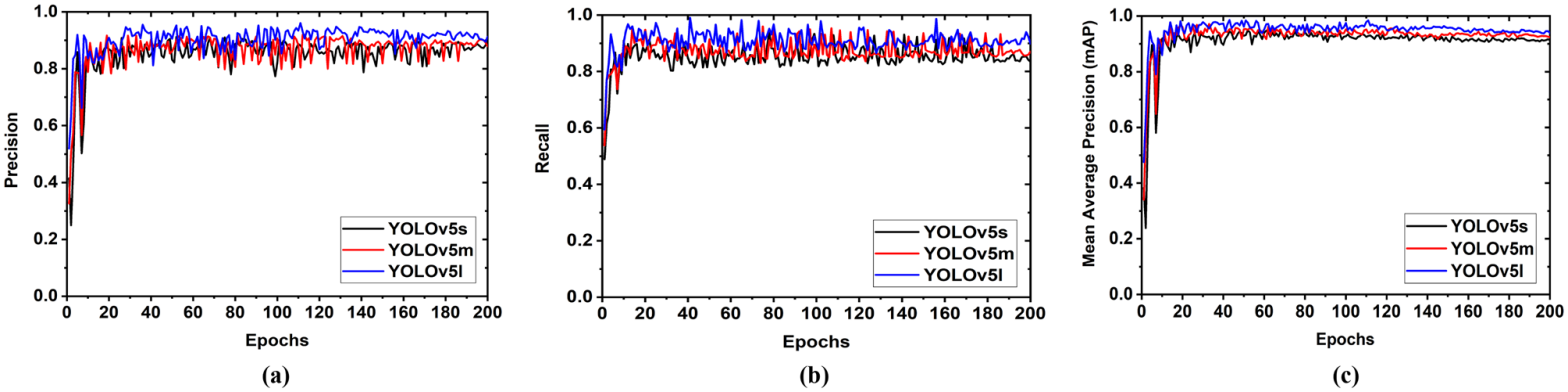
Tumor classification and detection analysis curve with respect to epochs: (**a**) Precision (P), (**b**) Recall/sensitivity (R), (**c**) Mean average precision(mAP).

We have investigated two tumor classes (i.e., benign and malignant) performance and detection performances through the YOLOv5s, YOLOv5m, and YOLOv5l models by using the RMW brain image training dataset. *Firstly,* it investigates the precision, recall, training classification loss, and validation classification loss performance with respect to epochs for the three versions of the YOLOv5 model. Precision is the ability of a model to classify and predict only the target tumor objects. The precision curve of YOLOv5s, YOLOv5m, and YOLOv5l models is illustrated in Fig. 14a, where the training dataset is utilized. Figure 14a shows that the precision rate is initially very low in all models, but it increases with respect to increasing the epochs. In addition, the precision rate of the YOLOv5s model is varied between 76 and 87%, whereas the rate of YOLOv5m is varied between 79 and 90%, and the rate of YOLOv5l is varied between 84 and 96%. As a result, the benign and malignant tumor classification and prediction rates of the YOLOv5l model are higher than the other two models.

On the other hand, recall is the ability of a model to find out all the relevant tumor classes. The recall curve of the three models is depicted in Fig. 14b, where the training dataset is employed. From Fig. 14b, it is examined that the recall rate is initially low (51% to 81%) for the YOLOv5s and YOLOv5m models, but it is 66% to 97% for the YOLOv5l model. It is also observed that the recall rate increases with respect to increasing the epochs. Moreover, the recall rate of the YOLOv5s model is varied between 81 and 91%, whereas the rate of the YOLOv5m is varied between 86 and 96%, and the rate of the YOLOv5l is varied between 90 and 98%. Thus, the target tumor classification and prediction performance of the YOLOv5l model is higher than the other two models. Besides, the mean average precision (mAP) for all models is observed, and it is shown in Fig. 14c. It is seen that the mAP of the YOLOv5l model is also higher than the YOLOv5m and YOLOv5s models. The overall investigation outcomes are presented in Table 5.

**Table 5:** Overall performance results of the different models.

Training models	Weight size	No. of training image dataset	Accuracy (A) (%)	Precision (P) (%)	Recall (R) (%)	Specificity (S) (%)	F1 score (%)	mAP (%)	Train classification loss	Validation classification loss
YOLOv5s	15 MB	1000	86.85	85.59	88.52	87.50	85.60	86.11	0.00568	0.0220
		2000	88.45	88.17	88.20	89.10	89.90	89.35	0.00543	0.0190
		3000	89.65	89.45	88.92	89.32	89.55	89.27	0.00522	0.0188
		3520 (80%)	91.77	91.15	90.97	91.61	91.65	91.20	0.00521	0.0185
YOLOv5m	43 MB	1000	89.62	89.17	88.48	89.57	88.92	88.29	0.00460	0.0191
		2000	90.66	90.41	89.98	90.23	90.19	90.12	0.00450	0.0185
		3000	91.25	91.13	91.15	91.18	91.12	91.10	0.00450	0.0171
		3520 (80%)	93.62	93.65	93.42	93.51	93.60	93.45	0.00442	0.0159
YOLOv5l	95 MB	1000	91.35	91.22	90.81	91.19	91.24	91.25	0.00331	0.0169
		2000	92.44	92.18	91.93	92.14	92.15	92.17	0.00320	0.0160
		3000	94.95	94.28	94.18	94.20	94.39	94.48	0.00313	0.0145
		3520 (80%)	96.32	95.17	94.98	95.28	95.53	96.12	0.00290	0.0130

**Figure 15:**
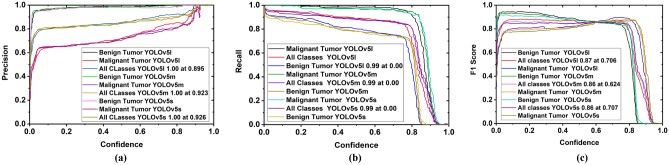
Benign and the malignant tumor classification performance curve with respect to confidence score: (**a**) Precision (P), (**b**) Recall (R), (**c**) F1 score.

*Secondly*, precision, recall, F1 score, and PR curve (Precision × Recall) are also investigated with respect to confidence score. The precision curve is illustrated in Fig. 15a. From Fig. 15a, it is observed that at the beginning, the precision is very low for all models, but the precision of benign tumor and malignant tumor classes are gradually increased with an increasing confidence score. Moreover, all class precision is 100%, when confidence scores are 0.926, 0.923, and 0.896 for YOLOv5s, YOLOv5m, and YOLOv5l models, respectively. So, it should be noted that all class's precision of YOLOv5l is higher at a reasonable confidence score (0.895) compared to other models. On the other hand, the recall curve is illustrated in Fig. 15b.

**Figure 16:**
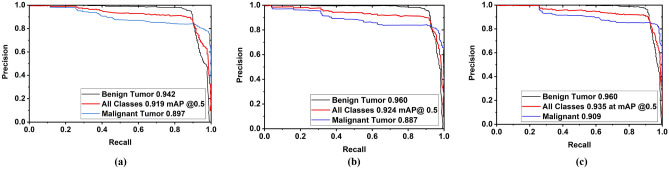
Precision × recall analysis curve for three models: (**a**) For YOLOv5s, (**b**) For YOLOv5m, (**c**) For YOLOv5l.

In Fig. 15b, it is examined that at the starting, the recall for benign and malignant tumor class is 99% for all models, but the recall is gradually decreased with respect to increasing confidence score up to 0.80. Although the recall of all classes for all models is 0% when confidence scores are between 0.85 to 1, the recall for the benign and malignant tumor class of the YOLOv5l is higher than that of the other two models. In addition, another parametric F1 score is examined. The F1 curve is depicted in Fig. 15c. The F1 score is calculated based on precision and recall. Figure 15c shows that F1 scores are 86%, 86%, and 87% in all classes, when confidences are 0.707, 0.624, and 0.706 for YOLOv5s, YOLOv5m, and YOLOv5l, respectively. Thus, it should be noted that the F1 score of all classes for the YOLOv5l is maximum at a moderate confidence score (0.706) compared to the other two models. Furthermore, the Precision × Recall curve is investigated to evaluate the performance of the tumor detection of the YOLOv5 models when the confidence score changes for each class. It helps to assess tumor prediction ability when the precision maintains a significant value with the increase in the recall. The Precision × Recall curve of YOLOv5s, YOLOv5m, and YOLOv5l is portrayed in Fig. 16a–c, respectively. It is observed from Fig. 16 that better performance outcomes are displayed closer to the right corner in the Figures. The cross values for benign tumor class are 94.2%, 96.0%, and 96.0% for YOLOv5s, YOLOv5m, and YOLOv5l models, respectively, whereas the cross values for malignant tumor class are 89.7%, 88.7%, and 90.9% for YOLOv5s, YOLOv5m, and YOLOv5l models, respectively. The mAP of all classes for YOLOv5s, YOLOv5m, and YOLOv5l models are 91.9%, 92.4%, and 93.5% at the rate of 0.5, respectively.

#### Tumor classification loss analysis

**Figure 17:**
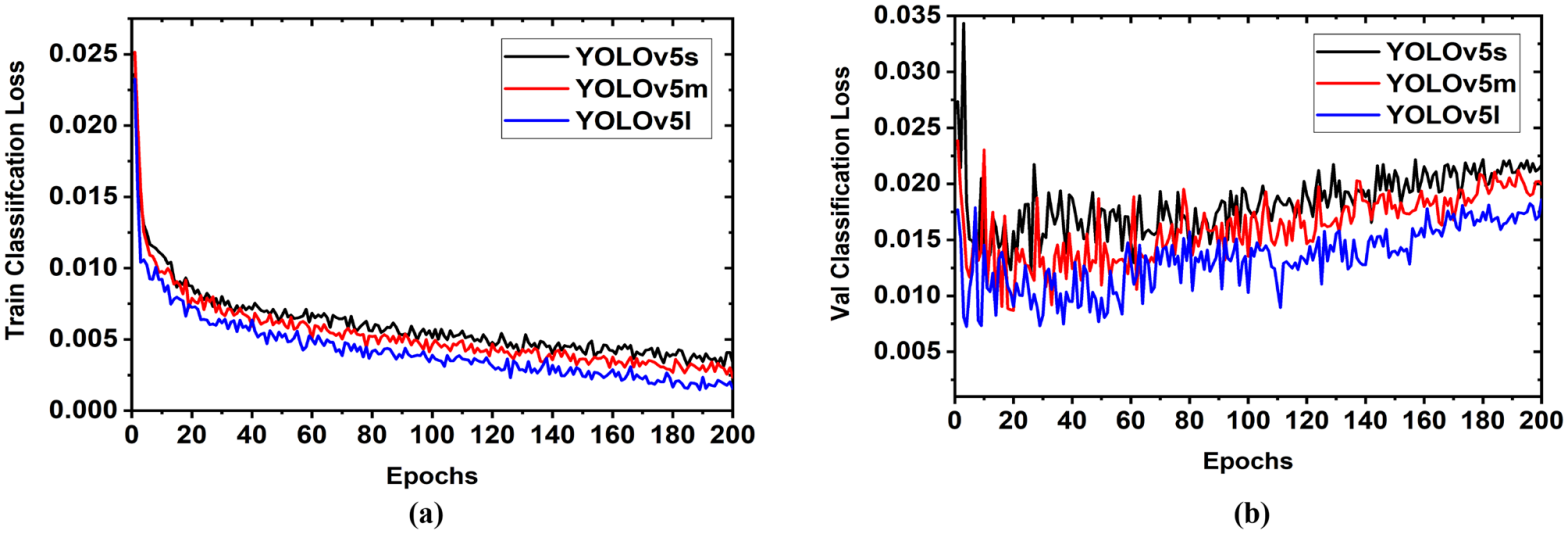
Tumor classification loss: (**a**) Training classification loss, (**b**) Validation classification loss.

The investigation of tumor class loss significantly predicts tumors' detection score in the RMW images. So, the tumor classification loss is investigated during the model's training. Two classification losses (i.e., training and validation classification losses) are observed by exploiting the image training dataset. The training classification loss of the tumor objects with respect to the epochs is illustrated in Fig. 17a. At the starting of the training phase, the training classification loss is relatively high (i.e., 0.25) for three models. But the training classification loss is gradually decreased when increasing the iteration. We used 200 epochs for training and validation classification in the investigation to get better classification outcomes. The training classification loss is comparatively high for the YOLOv5s model and low for the YOLOv5l model. After completing 200 epochs, the train classification losses are 0.004, 0.003,0.002 for YOLOv5s, YOLOv5m, and YOLOv5l, respectively. These incidences are found due to the variation of pre-trained weights of the YOLOv5 models. The best weight is used to train and test all models. The YOLOv5l model has large pre-trained weights from the other two models and takes slightly more time than other models to train the model. But the YOLOv5l model revealed a better result than other models.

In contrast, the validation classification loss of the tumor objects with respect to the epochs is illustrated in Fig. 17b. The validation classification loss is relatively high at the initial of the validation, such as 0.033 for YOLOv5s, 0.023 for YOLOv5m, and 0.016 for YOLOv5l models. However, after five epochs, it abruptly decreased to 0.014, 0.012, and 0.006 for YOLOv5s, YOLOv5m, and YOLOv5l, respectively. Afterward, it is also investigated that after completing 200 epochs, the validation classification losses are 0.022, 0.015, and 0.012 for the YOLOv5s, YOLOv5m, and YOLOv5l, respectively. But the losses for all models are slightly increased when increasing the epochs. It is noticeable that the YOLOv5l model showed better outcomes from the other two models. Finally, it is decided the YOLOv5l model exhibited better for tumor classification and detection results. The overall investigation outcomes are presented in Table 5.

#### Receiver operating characteristic (ROC) analysis

**Figure 18:**
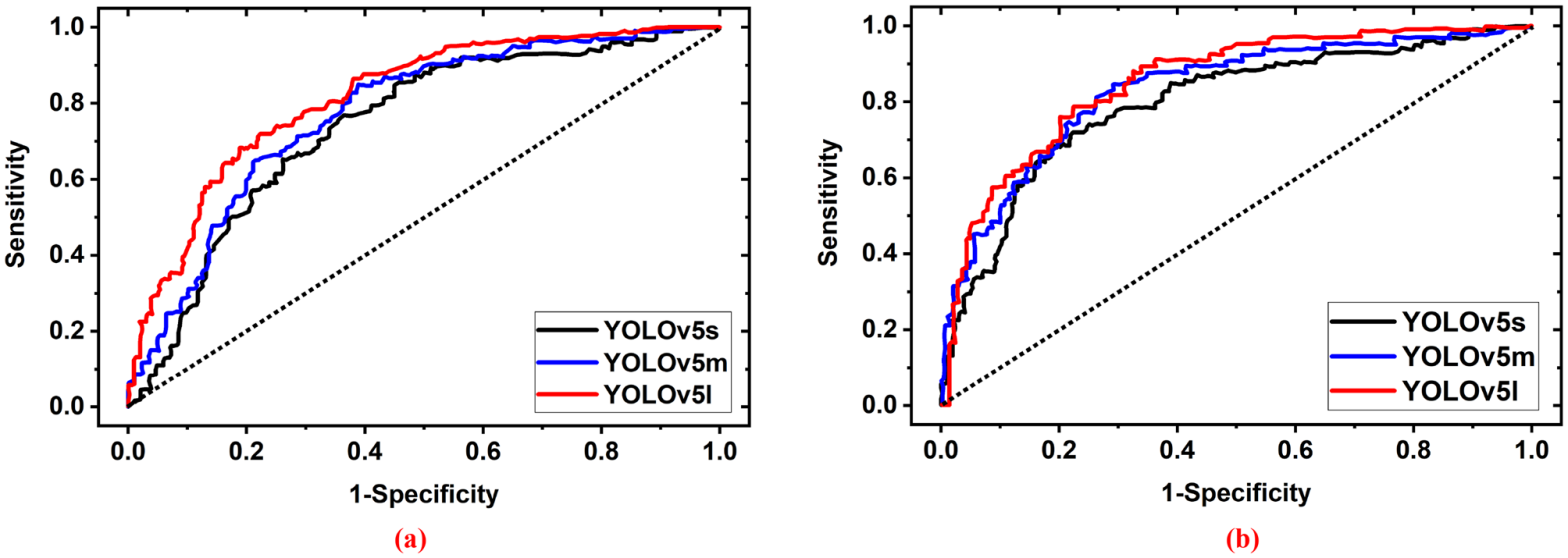
Receiver operating characteristic (ROC) curve with AUC for brain tumor detection and classification: (**a**) For benign tumor, (**b**) For malignant tumor.

The receiver operating characteristic (ROC) curve is an essential performance metric used in multi-class classification problems. The ROC is used to visualize the performance of a classification model across all thresholds. It also shows the capability of distinguishing between classes. The pixel-wise ROC curve with the area under the curve (AUC) for brain tumor detection and classification of three YOLOv5 models across all thresholds is depicted in Fig. 18. Figure 18a presented the ROC with AUC for benign tumor classification and observed that the YOLOv5l model performs better. The calculated AUC for benign tumor classification by the YOLOv5s, YOLOv5m, and YOLOv5l models are 81.56%, 83.25%, and 85.65%, respectively. Figure 18b presented the ROC with AUC for malignant tumor classification and observed that the YOLOv5l model performs better. The calculated AUC for malignant tumor classification of the YOLOv5s, YOLOv5m, and YOLOv5l models are 87.45%, 89.47%, and 91.36%, respectively. The overall investigation outcomes by the all models are presented in Table 5.

### Tumor classification and detection performances

**Figure 19:**
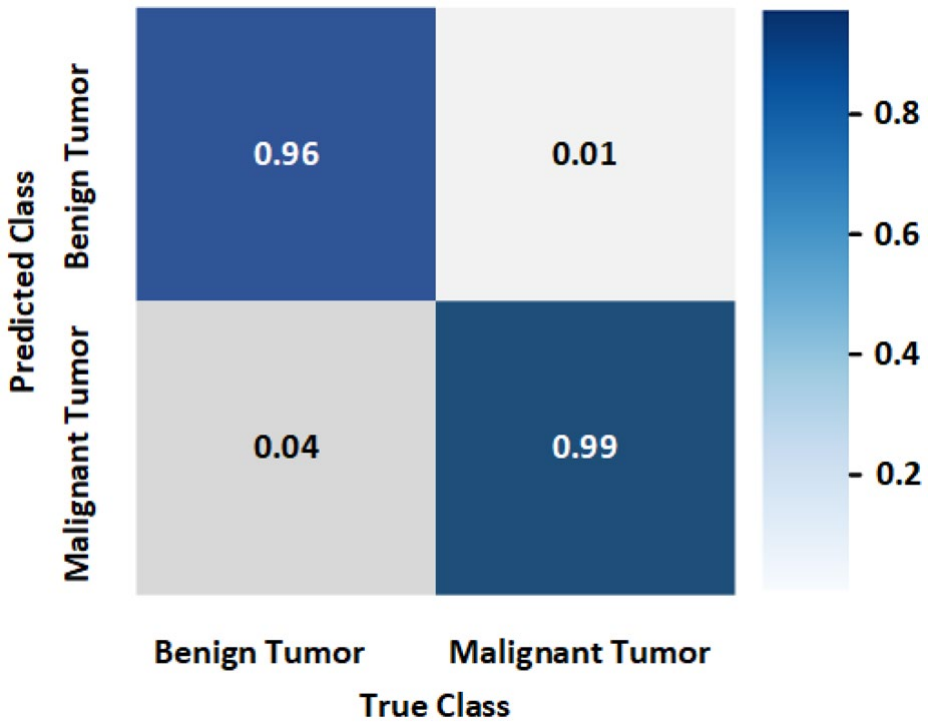
Confusion matrix of the YOLOv5l model.

**Figure 20:**
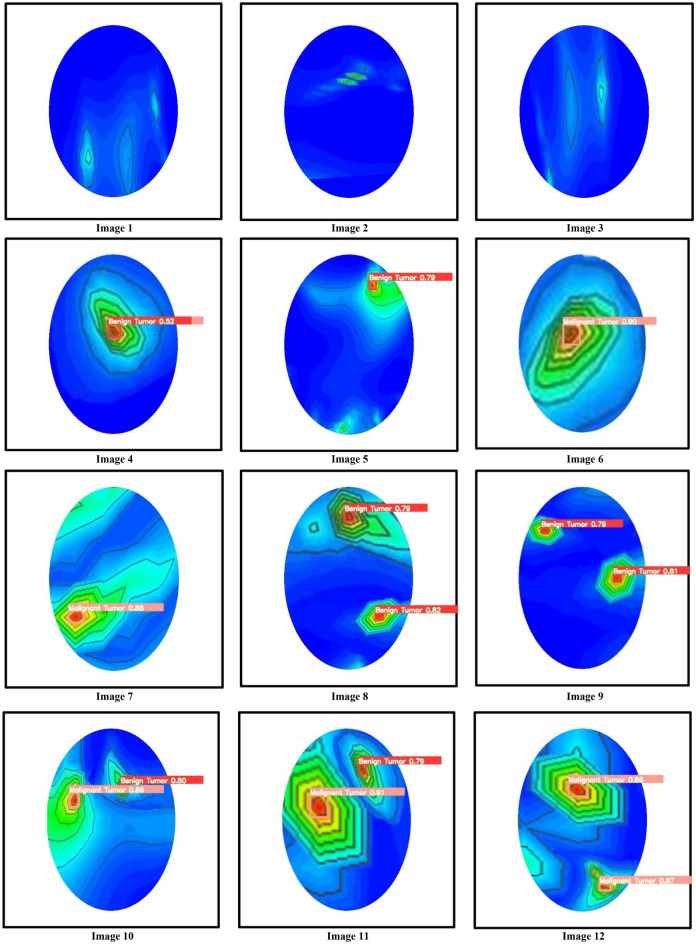
Tumor classification and detection results of the YOLOv5s model.

**Figure 21:**
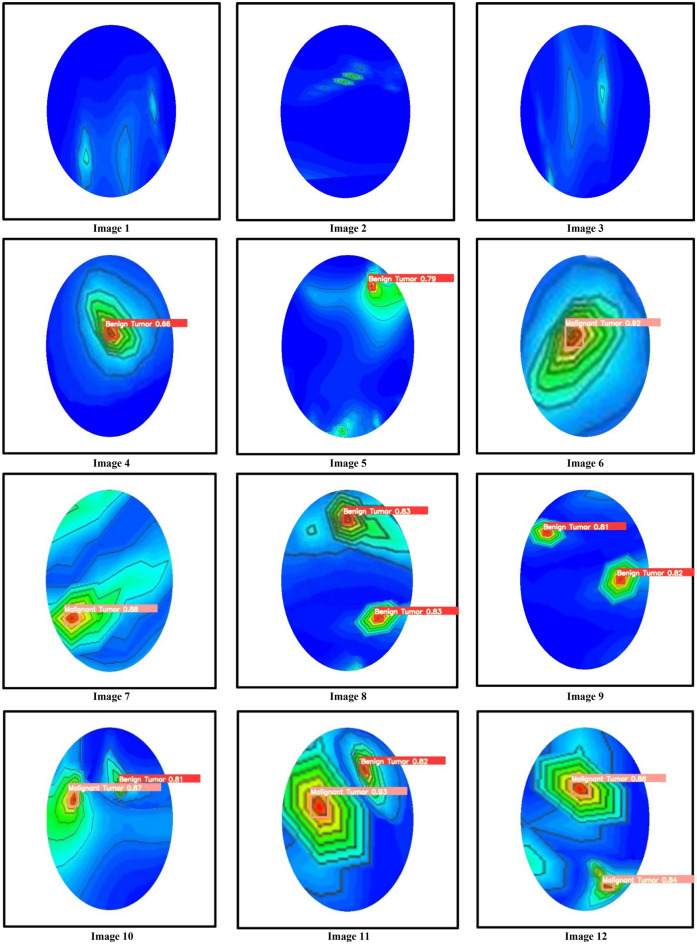
Tumor classification and detection results of the YOLOv5m model.

**Figure 22:**
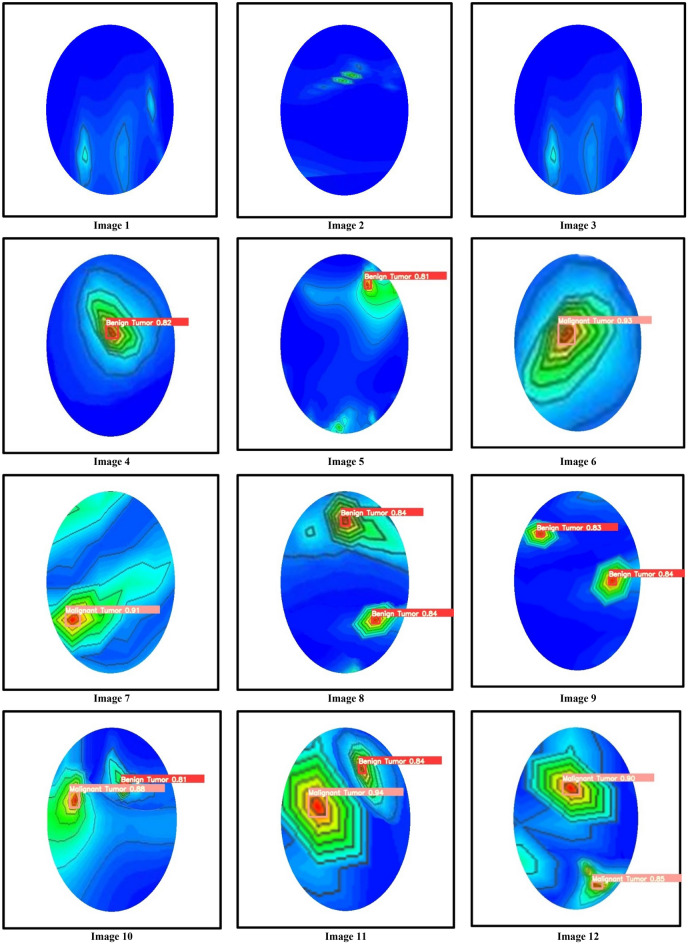
Tumor classification and detection results of the YOLOv5l model.

This section discusses the tumor classification and detection performances by the trained YOLOv5s, YOLOv5m, and YOLOv5l models. For performance investigation, we have been utilized our testing image dataset in different cases: (i) with one benign tumor, (ii) with one malignant tumor, (iii) with two benign tumors, (iv) with two malignant tumors, and (v) with one benign tumor and one malignant tumor. The confusion matrix of the best model YOLOv5l is presented in Fig. 19. It is realized from the confusion matrix; the model classified the tumor objects into two predicted classes and detected the benign and malignant tumors effectively in RMW brain images. In this investigation, 2080 benign and 1780 malignant tumor instances existed in the training dataset. It is observed from *the* confusion matrix 4% benign tumor out of 100% is miss classified as malignant tumors, and 1% *of* malignant tumors out of 100% is miss classified as benign tumors. The experimental tumor detection outcomes by the YOLOv5s, YOLOv5m, and YOLOv5l are depicted in Figs. 20, 21, and 22, respectively. For simplicity, the detection results of twelve images (non-tumor and tumor***-***based) are illustrated in this paper. All models have automatically detected the tumors accurately with a predicted bounding box including objectness score***,*** and classified the tumors into two classes (benign and malignant). But it is noticeable that the YOLOv5l model showed better performances than the YOLOv5s and YOLOv5m models. The detection accuracy of the YOLOv5l model has increased 2% to 30% compared to *the* other two models for different cases. The overall comparison results of YOLO models are presented in Table 6. The performance comparison results of YOLOv5l with the other five state-of-the-art object detection models are presented in Table 7. Finally, it is decided that the YOLOv5l model performs better than other YOLOv5 models and state-of-the-art models. This detection algorithm can be reliable in microwave head imaging systems to automatically detect and classify tumors.

**Table 6:** Overall comparison of tumor detection and classification with objectness score by the YOLOv5 models.

Image no.	Tumor classification				Objectness detection score		
	Benign tumor 1	Benign tumor 2	Malignant tumor 1	Malignant Tumor 2	YOLOv5s	YOLOv5m	YOLOv5l
1	No	No	No	No	No score	No score	No score
2	No	No	No	No	No score	No score	No score
3	No	No	No	No	No score	No score	No score
4	Yes	No	No	No	0.52	0.66	0.82
5	Yes	No	No	No	0.79	0.79	0.81
6	No	No	Yes	No	0.90	0.92	0.93
7	No	No	Yes	No	0.86	0.88	0.91
8	Yes	Yes	No	No	0.79 and 0.82	0.83 and 0.83	0.84 and 0.84
9	Yes	Yes	No	No	0.79 and 0.81	0.81 and 0.82	0.83 and 0.84
10	Yes	No	Yes	No	0.80 and 0.86	0.81 and 0.87	0.81 and 0.88
11	No	Yes	No	Yes	0.79 and 0.91	0.82 and 0.93	0.84 and 0.94
12	No	No	Yes	Yes	0.86 and 0.87	0.88 and 0.84	0.90 and 0.85

**Table 7:** Performance comparison outcomes of the YOLOV5l and state-of-the-art object detection models.

References	Model Name	Accuracy (A) (%)	Precision (P) (%)	Recall/Sensitivity (R) (%)	Specificity (S) (%)	F1 score (%)
^44^	Faster R-CNN	91.96	91.45	90.95	91.87	91.39
^45^	Mask R-CNN	91.72	91.58	90.86	91.37	91.38
^47^	F-CNN	91.97	91.18	90.45	90.65	90.49
^46^	Residual R-CNN	89.96	89.68	89.69	89.15	89.28
^14^	YOLOv3	92.97	92.75	92.67	92.81	92.89
Proposed	YOLOv5l	95.32	95.17	94.98	95.28	95.53

**Figure 23:**
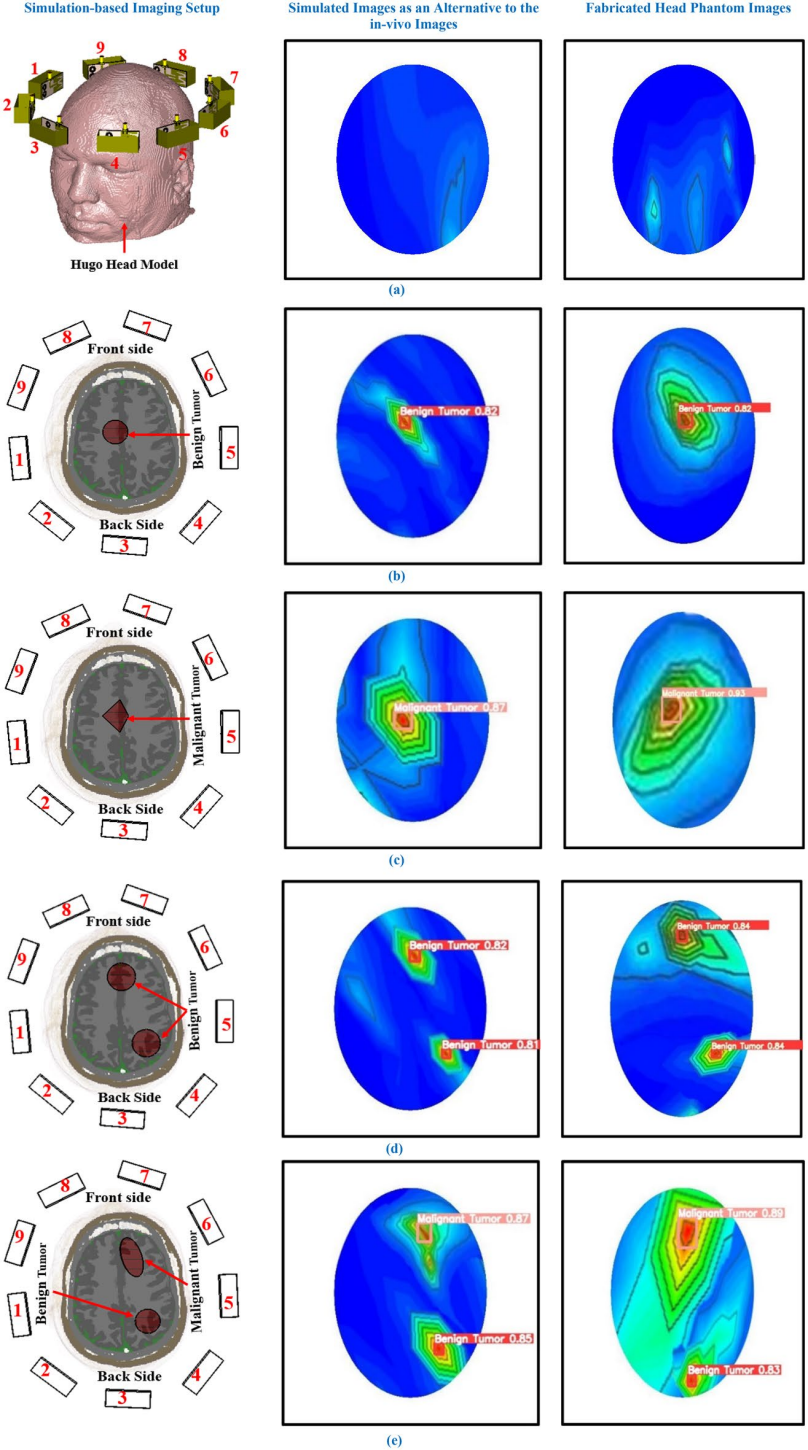
Differences between phantom images and simulated images as an alternative to the in-vivo images: (**a**) Non-tumor images, (**b**)With a single benign tumor, (**c**) With a single malignant tumor, (**d**) With two benign tumors, (**e**) With one benign and one malignant tumor.

This research has generated the head phantom images and investigated tumor detection performance through the YOLOv5 algorithms. Since the proposed imaging system can be reliable in real-time application due to its portability, non-invasive, safety, and low-cost features. Practically, the real data will be collected to produce brain images from the system. After that, the tumor(s) will be automatically identified and classified through the YOLOv5l algorithm in a real-time application. Thus, it is necessary to investigate whether the YOLOv5l algorithm will detect the tumor in in-vivo images (i.e., real images) instead of phantom images. However, the clinical trial is necessary to inspect the image generation and detect the tumors in an actual application. Due to covid-19 pandemic and clinical trial permission limitation during research, we could not perform the physical experiment to create real images called in-vivo images. But we have analyzed the real brain tissue-mimicking head called “Hugo” model with the antenna array through the simulation via CST-2019 software instead of physical experiment. The head model is imported from the CST 2019 software. The simulated performances instead of a real investigation can be reliable to the in-vivo collected images. The Hugo head model consists of six brain tissue layers acting as the real brain tissues. The permittivity and conductivity of the layers are the same as the real brain tissues^50,51^. The tumor(s) are placed in different locations with different sizes and shapes in the “Hugo” model to examine the image generation performance. All benign and malignant tumor(s) are placed at 60 mm depth from the top of the head model. The considered diameters of the benign tumor are D = 10 mm,12 mm,13 mm, and 14 mm, with circular shapes. On the other hand, the considered lengths of the malignant tumor are L = 15 mm with triangle-shaped, and 18 mm with an elliptical shape. The electrical properties of the Hugo head tissue (i.e., considered as a real head)^18,19^ and fabricated head phantom tissues are presented in Table 8. The overall simulation-based imaging system with a Hugo head model including tumor(s) and nine antenna array is presented in Fig. 23. However, simulated data as an alternative to real data are collected from the simulation system, and then an image reconstruction algorithm is applied to generate brain images. After that, the pre-trained YOLOv5l algorithm’s weights were applied for testing and detecting the tumors in the simulated reconstructed brain images. The difference between resultant simulated images (i.e., the alternative to the in-vivo image) and phantom images are illustrated in Fig. 23. It is observed that both images are almost similar with tumor locations. The proposed YOLOv5l has been detected the tumor(s) with appropriate locations including bounding box in both images. So, from the comparison results, we can realize that the proposed YOLOv5l model can be a reliable to detect the tumors in collected in-vivo images.

**Table 8:** Electrical properties of brain tissues in Hugo head model and fabricated head phantom model.

Name of the tissues	Hugo head model		Fabricated head phantom model	
	Relative permittivity at 2 GHz	Conductivity at 2 GHz	Relative permittivity at 2 GHz	Conductivity at 2 GHz
Dura	52.12	1.23	47.93 ± 1.523	1.66 ± 0.003
CSF	68.64	2.413	63.78 ± 2.041	2.719 ± 0.412
Gray matter	52.73	0.942	47.74 ± 1.023	1.38 ± 0.012
White matter	38.89	0.591	35.98 ± 1.245	0.765 ± 0.045
Benign tumor	25.45	0.568	22.65 ± 1.222	0.970 ± 0.122
Malignant tumor	68.14	3.956	64.05 ± 2.107	4.18 ± 0.074

## Conclusion

A deep learning-based YOLOv5 object detection model is employed in portable microwave head imaging (MWHI) system to automatically detect and classify brain abnormalities. A 3D wideband nine antenna array and tissue-mimicking head phantom with the non-tumor, benign and malignant tumor are used in the developed MWHI system to reconstruct the brain images and verify the system performances. The generated image dimension is 640 × 640 pixels. Four hundred sample images with non-tumor and tumor(s) are collected from the developed MWHI system. After that, images are pre-processed and augmented to create a training dataset consisting of 4400 images and then utilized for training, validation, and testing the YOLOv5s, YOLOv5m, and YOLOv5l network models. The model consists of CoBL module, BCSP module, Leaky ReLu activation function, and many 3 × 3 and 1 × 1 convolutional layers. These modules and functions optimize the network, reduce the inference time, increase computational speed, and diminish the size of the feature map on three scales. Three scales are responsible for detecting a small, medium, and large sized tumor in the RMW images. The models are investigated by employing a testing dataset and verifying the outcomes. The YOLOv5l model performed better than the YOLOv5s, YOLOv5m, and state-of-the-art object detection models regarding tumor classification and detection. The YOLOv5l model showed high accuracy, precision, recall, specificity, F1 score, and mAP with low loss. However, the Yolov5l model effectively detected the target tumor(s) with an appropriate location with a bounding box and scored and classified tumors into benign and malignant classes. Finally, it is decided that the YOLOv5l model can be reliable for automatic tumor detection and classification in microwave brain imaging systems.
